# Systemic Lactate Acts as a Metabolic Buffer in Humans and Prevents Nutrient Overflow in the Postprandial Phase

**DOI:** 10.3389/fnut.2022.785999

**Published:** 2022-03-10

**Authors:** Lisa Schlicker, Gang Zhao, Christian-Alexander Dudek, Hanny M. Boers, Michael Meyer-Hermann, Doris M. Jacobs, Karsten Hiller

**Affiliations:** ^1^Department for Bioinformatics and Biochemistry, Braunschweig Integrated Centre of Systems Biology (BRICS), Technische Universität Braunschweig, Brunswick, Germany; ^2^Deutsches Krebsforschungszentrum, Heidelberg, Germany; ^3^Department of Systems Immunology and Braunschweig Integrated Centre of Systems Biology, Helmholtz Centre for Infection Research, Brunswick, Germany; ^4^Centre for Individualised Infection Medicine, Hanover, Germany; ^5^BRENDA Enzyme Database, BRICS, Technische Universität Braunschweig, Brunswick, Germany; ^6^Unilever Foods Innovation Centre, Wageningen, Netherlands; ^7^Institute of Biochemistry, Biotechnology and Bioinformatics, BRICS, Technische Universität Braunschweig, Brunswick, Germany

**Keywords:** metabolic flux, glucose, wheat, stable isotope labeling, metabolic modeling, lactate

## Abstract

On an organismal level, metabolism needs to react in a well-orchestrated manner to metabolic challenges such as nutrient uptake. Key metabolic hubs in human blood are pyruvate and lactate, both of which are constantly interconverted by very fast exchange fluxes. The quantitative contribution of different food sources to these metabolite pools remains unclear. Here, we applied i*n vivo* stable isotope labeling to determine postprandial metabolic fluxes in response to two carbohydrate sources of different complexity. Depending on the ingested carbohydrate source, glucose or wheat flour, the net direction of the lactate dehydrogenase, and the alanine amino transferase fluxes were adjusted in a way to ensure sufficient availability, while, at the same time, preventing an overflow in the respective metabolite pools. The systemic lactate pool acts as a metabolic buffer which is fueled in the early- and depleted in the late-postprandial phase and thus plays a key role for systemic metabolic homeostasis.

## Introduction

When a healthy individual in fasting state is challenged with a nutritional intervention such as a meal, the metabolism is confronted with an excessive supply of nutrients causing metabolite levels in the blood to increase and homeostasis to be out of balance. Systemic metabolism has to rapidly undergo complex adaptions to switch from the former catabolic to the anabolic state to keep the systemic concentrations of food-derived nutrients including glucose, amino acids and fatty acids in a physiological range (1–3). The goal to understand or even predict postprandial metabolism, has been approached by nutritional research in several ways. Metabolomics emerged as a popular tool in recent years (4–6), however, the interpretation of changing metabolite levels often remains descriptive and mechanistic insights are difficult to derive (7, 8). On the other hand, complex isotopic labeling experiments employing dual or even triple tracer based approaches determine highly accurate metabolic flux information and thus provide a better mechanistic understanding, but are usually limited to the kinetics of single metabolites, in most cases glucose (9–11). To gain quantitative flux information of an entire metabolic network in the postprandial phase, a setting in which steady-state conditions do not apply, remains challenging. Current approaches rely on a combination of stable isotope labeling and time-resolved sampling together with suitable data analysis tools (8, 12, 13).

Here, we applied an instationary ^13^C metabolic flux analysis (INST-^13^C-MFA) approach to investigate differences in metabolic fluxes downstream of glucose in response to two different carbohydrate sources. In a randomized, cross-over nutritional intervention study, 12 healthy subjects consumed either a glucose drink supplemented with 2% fully ^13^C-labeled glucose (referred to as GLC) or a wheat porridge supplemented with 2% fully ^13^C-labeled wheat flour (referred to as WP). The content of available carbohydrates was matched to 50 g in both interventions. Due to the low amount of the initial ^13^C labeling and thus even lower enrichment of downstream plasma metabolites, we employed a specialized GC-MS-based analytical workflow for isotopic enrichment quantification (14). We calculated time-resolved, quantitative isotopologue concentrations and applied an ordinary differential equation (ODE) model of central carbon metabolism to determine postprandial metabolic fluxes and to identify intervention-induced flux alterations.

Our results demonstrate that human metabolism adjusts metabolic fluxes in a distinct and carbohydrate type specific manner. By analyzing the ^13^C-enrichment in plasma metabolites, we were able to observe the integrated net results of the metabolic activities of all tissues providing a more systemic understanding of the postprandial phase as compared to glucose fluxes alone.

## Materials and Methods

### Study Design

The study was conducted by the Quality Performance Service Netherlands B.V., Groningen, The Netherlands, a clinical research organization (CRO) according to the principles of Good Clinical Practice, the Declaration of Helsinki (2008). Ethics approval was obtained from the Medical Ethics Committee of Brabant, Tilburg, the Netherlands. The presented trial was registered at www.clinicaltrials.gov as NCT02662738. The presented results represent the secondary outcome measures 1. Total blood glucose, 2. Insulin, 5. ^13^C Metabolites of glucose and 6. ^12^C Metabolites of glucose.

#### Subjects

In total, 12 healthy male subjects in the age between 20 and 50 with a fasting glucose value between 3.4 and 6.1 mmol/l and a BMI between 18.0 and 25.0 kg/m^2^ participated and completed the study. For further inclusion/exclusion criteria and ethical aspects refer to Supplementary Methods S1.

#### Experimental Design

The study used a randomized, controlled, full cross-over (within subject) design. Subjects attended the initial screening day followed by three test days, at least 1 week apart. Participants consumed a standardized evening meal at the CRO. All participants fasted 10 h prior to ingestion of the test product but were allowed to drink water until 1 h before consumption of the intervention. Since the primary outcome measure of this study was time-resolved glucose kinetics using the dual labeled isotope technique, a priming dose of ^2^H-labeled glucose was administered as a bolus (5.6 mg/kg body weight) followed by a continuous infusion of deuterated glucose for 8 h (0.07 mg/kg body weight). Two hours after the start of the infusion, subjects consumed the labeled test products within 15 min (with 100 ml tap water in case of WP). This manuscript focuses on the orally administered tracers glucose and labeled wheat flour and the resulting ^13^C-labeled downstream metabolites. The data of the intravenous tracer are not relevant for the secondary outcome measures and therefore not further evaluated.

#### Test Product and Preparation

The study was conducted with three food products, however, we focused during data analysis on two test products only. The wheat porridge (WP) was made from commercially available standard wheat flour (Type B German wheat: 70 ± 0.5 g of wheat flour ≈ 50 g available carbohydrates) of which 2% consisted of ^13^C-universally-labeled wheat flour very-closely related to type B German wheat flour. The ^13^C-labeled wheat flour was grown under ^13^CO_2_ atmosphere, therefore all biomolecules including starch, protein, lipids and metabolites are fully ^13^C-labeled (~97% ^13^C enrichment). Further details on the production and testing of the ^13^C-labeled wheat flour is provided in Supplementary Method S2. GLC consisted of a solution of ^13^C-universally-labeled glucose (2%, 1 g) and 49 g unlabeled glucose in 250 mL water. The amount of available carbohydrates was matched to 50 g in WP and GLC with 2 atom % excess in both interventions.

#### Treatment Assignment

All subjects were randomly allocated to the study treatments (full cross-over design with three periods based on a Williams design), such that the order of consumption of the treatments is balanced. The randomization code was kept strictly confidential and randomization was performed by Unilever. The randomization code and the randomization list were generated using SAS software (Version 9.4).

#### Sample Collection

Blood samples were collected in K_2_-EDTA-tubes containing dipeptidyl peptidase-IV (BD Diagnostics) at time points −60, −30, −5, 15, 30, 45, 60, 75, 90, 105, 120, 150, 180, 210, 240, 270, 300, 330, and 360 min and centrifuged at 1,300 g for 10 min at 4°C within 30 min after collection to limit pre-analytical variation. Resulting plasma was stored at −80°C prior extraction.

### Analytical Techniques

#### Metabolite Extraction From Plasma

Prior to metabolite extraction, plasma samples were thawed on ice for 30 min. Extraction was performed in technical triplicates as reported before (15). Briefly, we combined 20 μl plasma with 180 μl extraction fluid (4+1, methanol:water) containing three internal standards [Pentanedioic acid-d6 (final concentration: 20 μM), U^13^C-Ribitol (final concentration: 12.7 μM), Norleucine (final concentration: 10 μM)]. The mixture was subsequently mixed at 4°C and 1,400 rpm for 5 min and centrifuged at 4°C and 21,000 g for 10 min. After centrifugation, 140 μl of the supernatant were transferred to a glass vial with insert and left-over extract was collected, mixed and used for the preparation of pool samples. The extracts were dried overnight at −4°C in a refrigerated vacuum concentrator (Labconco, Kansas City, USA) and stored at −80°C prior to GC-MS measurement.

#### Metabolite Derivatization and GC-MS Measurement

Sample derivatization and GC-MS measurement were performed according to Krämer et al. (14). Briefly, metabolites were derivatized with 15 μl of 2% methoxyamine hydrochloride in pyridine for 90 min and 15 μl N-methyl-N-trimethylsilyl-trifluoroacetamide (MSTFA) for additional 30 min at 40°C under continuous shaking using a PAL RTC autosampler.

For GC-MS selected ion monitoring (SIM) data acquisition, we used the method reported by Krämer et al. (14) with slight adaptions. We operated an Agilent 7890A GC equipped with a 20 m DB-5MS capillary column (0.18 mm inner diameter, 0.18 μm film thickness) connected to an Agilent 5977B High Efficiency source (HES) to record the data. To obtain good peak shapes for highly abundant metabolites such as glucose but to also cover low abundant metabolites, we injected the samples twice with different split ratios (highly abundant−50:1, low abundant−20:1).

#### Insulin Data

Insulin was measured by a chemiluminescent microparticle immunoassay (The ARCHITICT® insulin assay, Abbott, Laboratories, Abbott Park, USA).

### Data Analysis

Obtained SIM GC-MS data were processed using the software package MetaboliteDetector (16) as described by Krämer et al. (14).

#### Determination of Mass Isotopomer Distributions

Mass isotopomer distributions (MIDs) were determined from the generated SIM data using MetaboliteDetector's sum formular-based MID wizard (16). For the mass isotopomers of the metabolites glycine, citrate, glutamine, and glutamate, the non-labeled spectra-based correction was applied (17). Raw data are uploaded online (https://doi.org/10.24355/dbbs.084-202109291537-0) and are presented in Supplementary Table S1.

#### Absolute Quantification

We applied MetaboliteDetector's batch quantification wizard for the relative quantification of the metabolite levels for the SIM data by summing up the intensities of all m/z recorded per metabolites (16). The relative quantities were transformed into absolute concentrations using the external calibration curves which were run in triplicates in every sample batch. Standards for external calibration were prepared in MQ water at six different concentration steps per metabolite according to Supplementary Table S2 and extracted similar to the plasma samples (see section Metabolite Extraction From Plasma). For selection of suitable concentration ranges, commonly observed blood metabolite concentrations were taken into account (www.hmdb.ca) (18). The quality of the calibration curves was evaluated by the obtained *R*^2^ values for each metabolite separately. Raw data are uploaded online (https://doi.org/10.24355/dbbs.084-202109291537-0) and are presented in Supplementary Table S3.

#### Contribution of Food-Derived Isotopologues to the Metabolite Plasma Pool

The contribution of food-derived labeled metabolites to the plasma pool was calculated by dividing the isotopologue concentration (*c*_iso_) at maximum by the corresponding plasma concentration of the metabolite (*c*_met_). To account for the amount of labeling (2%), the result was scaled with 50. To obtain the contribution in percent, the results were multiplied by 100 according to Equation (1).


(1) $$\begin{array}{l} Contribution\left[\%\right]=\frac{c_{iso}}{c_{met}}\;*\;50\;*\;100 \end{array}$$

### Statistical Analysis

#### Outlier Analysis and Data Exclusion

Outliers present in the MID and the absolute concentration data sets varying more than 1.5 ^*^IQR (interquartile range) were identified and removed using R, version 3.4.4. Out of 12 subjects, the data sets of 11 subjects were included in the further analysis. Subject 02 was excluded due to technical failures.

#### Repeated Measures (rm)ANOVA

Repeated measures ANOVA (rmANOVA) was performed using R version 3.4.4. For the absolute concentrations, the MID data set, the product of absolute concentration and ^13^C-enrichment (referred to as *isotopologue concentrations*) as well as the insulin data, rmANOVA was performed on the medians of the technical triplicates on the factors intervention and time and the combination of both. A value of *p* < 0.05 was considered statistically significant.

### Metabolic Network Model

The network model construction and subsequent fitting were described earlier and applied to this data set with slight adaptions (15).


(2) $$\begin{array}{l} \frac{dC_{pyr}}{dt}=k_{GLY}C_{glc}+k_{LDHb}C_{lac}+k_{ALTb}C_{ala}-(k_{LDHf}+k_{ALTf}\\ \;+k_{TCA}+d_{pyr})C_{pyr}\\ \frac{dC_{lac}}{dt}=k_{LDHf}C_{pyr}-\left(k_{LDHb}+d_{lac}\right)C_{lac}\\ \frac{dC_{ala}}{dt}=k_{ALTf}C_{pyr}+k_{Pro}C_{pro}-\left(k_{ALTb}+d_{ala}\right)C_{ala}\\ \frac{dC_{cit}}{dt}=k_{TCA}C_{pyr}-d_{cit}C_{cit} \end{array}$$

where *k* represents the rate of intracellular transformation (see **Figure 3A**), *C* represent the metabolite concentrations and *d* represents the intracellular processes that are not explicitly modeled; subscripts denote metabolites (Equation 2). The kinetics of glucose M6 (*C*_glc_) and protein (*C*_pro_) were linearly interpolated from the measured data and directly fed to the model. The rest of the metabolites (*C*_pyr_, *C*_lac_, *C*_ala_, and *C*_cit_) were simulated. The protein data (*C*_pro_) were interpolated from the amino acids glutamate M5, glutamine M5, valine M5, and threonine M4, by taking into account the abundance of these amino acids in mol% in the wheat protein gluten (19) according to Equation (3).


(3) $$\begin{array}{l} Protein\left[\mu M\right]=\frac{\left(Glu_{M5}+Gln_{M5}+Val_{M5}+Thr_{M4}\right)\;*\;100}{\left(31.9+5.4+2.8\right)} \end{array}$$

In order to compare model output (intracellular concentration) with the measured concentrations in blood, a time-resolved relationship between blood and intracellular metabolite concentrations was estimated by taking into account values reported in literature as well as data generated in this study (Supplementary Tables S4, S5), as:


(4) $$\begin{array}{l} \frac{C_{b}}{C_{i}}=L\left(\left[0,120,360\right],\left[s_{pa},s_{a},s_{pa}\right],time\right) \end{array}$$

where subscripts *b* and *i* denote blood and intracellular compartments, respectively, *L* denotes linear interpolation, subscripts *pa* and *a* denote post-absorptive or absorptive, respectively (Equation 4). Here, for the scaling factors, the separation between the absorptive and the post-absorptive phase was assumed to be 120 min. This assumption set the stage for the following modeling study, where the optimal separating time for blood metabolites was determined [see below (2)].

The data set subjected to modeling was generated by averaging over the median value of the three replicates of each subject at each time point. The metabolic network analysis was composed of the following steps:

(1) The network model was fitted to the data of both interventions simultaneously, with the side condition that the ratio between parameter associated with the two interventions is limited in [0.1, 10]. The ratio between *k*_GLY_ and *k*_Pro_ was also limited in the same range. No qualitative changes of the result were detected if this limit was set to [0.3, 3.3]. The fitting procedure was guided by minimizing the root mean square (RMS) difference between (scaled) model output (y) and the measured data point ($\hat{\text{y}}$) referred to as “cost” throughout the manuscript (Equation 5).
(5) $$\begin{array}{l} RMS=\sqrt{\sum_{i=1}^{n}\frac{{\left(y_{i}-\hat{y_{i}}\right)}^{2}}{\sigma_{i}^{2}}} \end{array}$$
  where σ denotes the standard deviation, and *n* denotes the number of data points. A differential evolution-based global optimizer, configured to favor local search, was employed for fitting. The fitting was repeated 50 times, among which the best result served as baseline for further analysis.
(2) To identify appropriate time windows for the absorptive and post-absorptive phase, the data were separated into two consecutive time windows. We investigated the separating time points 75, 90, 105, 120, and 150 min. For example, with the separating point 120 min, the whole window was separated into [0, 120] min and [120, 360] min. The fitting procedure described above was repeated in the early and late window, respectively. For every investigated separating point, it was first confirmed that the sum of the best costs in the two windows (obtained in step 2) was lower than the best cost of the entire window (obtained in step 1) (Supplementary Table S6). This served as a sanity check for separating the whole window into two separate ones. The sum of the best costs in the two separate windows was then compared for different separating points (see Supplementary Table S6). The lowest sum of cost was regarded as indicative of an appropriate time for separating the absorptive and post-absorptive phase.
(3) To determine differences between the two interventions, GLC and WP, the distribution of the transformation rates, i.e., *k* and *d* in Equation (2) were sampled using a differential evolution based adaptive Markov Chain Monte Carlo (MCMC) algorithm (*n* = 10,000). The resulting parameter populations were fed to a logistic regression model. The performance of the logistic regression model was evaluated by the area under the curve (AUC) of the receiver operator curve (ROC). An AUC-ROC of 0.75 and higher was considered indicative for significant differences between GLC and WP, and the analysis was performed for the early and the late window separately.
  The generated set of parameters was transferred into postprandial metabolic fluxes according to Equation (6.1) (for the early time window 0–90 min) or Equation (6.2) (for the late time window 90–360 min).
(6.1) $$v=k\;*\;median\left(C_{met}(0-90\min)\right)\;*\;(\frac{1}{s_{a}})\;*50$$
(6.2) $$\begin{array}{l} v=k\;*\;median\left(C_{met}(90-360\min)\right)\;*\;(\frac{1}{s_{a}})\;\\ \;*\;50 \end{array}$$
  where *v* denotes flux, *k* denotes the respective parameter, *C*_met_ denotes the median concentration of the respective isotopologue and time window and *s*_a/pa_ denotes the corresponding scaling factor. Fluxes were multiplied by 50 to account for 2% of labeling of the oral tracers. Ninety percent credible intervals were calculated using R (version 3.4.4).

## Results and Discussion

In this study, we aimed for the quantitative determination of metabolic fluxes downstream of glucose in the dynamic postprandial phase in human subjects. For this purpose, we combined *in vivo* stable isotope labeling, time-resolved plasma sampling and absolute quantification of metabolites with an ODE-based metabolic modeling approach. We investigated and compared the postprandial central carbon metabolism of two different carbohydrate sources, either glucose (GLC) or wheat porridge (WP) by matching total carbohydrate intake (50 g) and the amount of isotopic enrichment of the carbohydrate sources (2%). For the GLC intervention, all subjects consumed the glucose equivalent of 50 g available carbohydrates of which 2% were uniformly ^13^C-labeled ([U^13^C_6_] glucose). For the WP intervention, the subjects consumed an equivalent amount of carbohydrates based on porridge prepared from wheat flour of which 2% were substituted by fully ^13^C-enriched wheat flour.

### WP Intake Alters the Postprandial Insulin but Not Glucose Response

**Figure 1:**
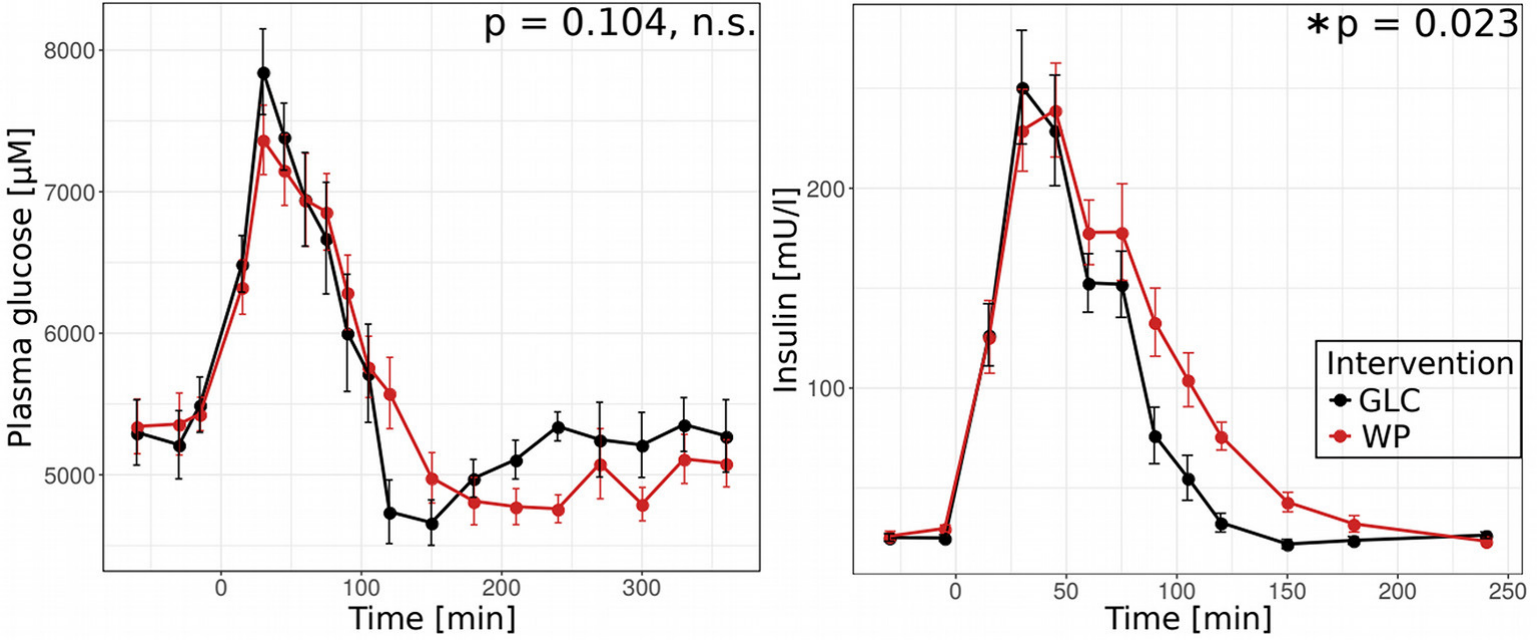
WP intake alters insulin response. Postprandial blood glucose (left) and insulin (right) after intake of glucose (GLC—black) and wheat porridge (WP—red); average of 11 study subjects ± standard error of the mean (SEM), statistics: rmANOVA.

In a first step, we compared postprandial glucose concentrations and insulin levels for both interventions, GLC and WP (Figure 1). After GLC intake, the insulin profile closely followed that of blood glucose, reaching maximum glucose and insulin concentrations of 7,800 μM and 250 mU/l after 30 min, respectively. Following WP intake, the maximum glucose concentration was also reached after 30 min, but tended to be lower (7,400 μM) and the highest insulin concentration of 240 mU/l was reached 45 min after food intake. While the glucose profiles obtained for GLC and WP were not significantly different, we observed clear differences in the declining phase of the insulin profiles. Insulin levels decreased significantly faster after intake of GLC and were back to baseline after 150 min. In contrast, baseline insulin levels were reached 240 min after WP intake indicating differences in the postprandial responses.

### ^13^C Metabolic Flux Analysis of the Postprandial Phase *in vivo*

**Figure 2:**
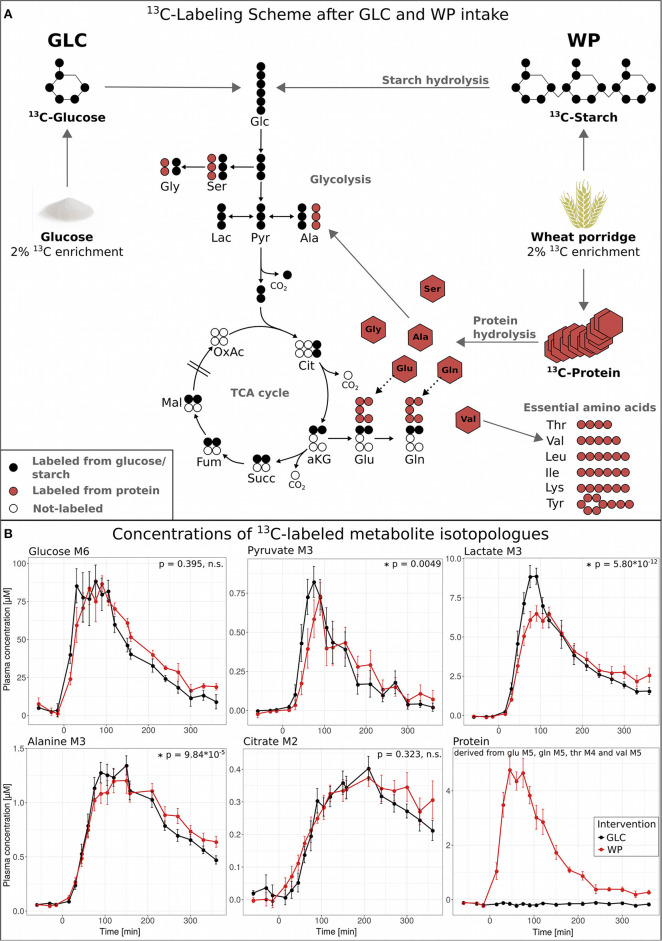
^13^C metabolic flux analysis in the postprandial phase. **(A)** Overview of ^13^C tracer metabolism for both interventions: GLC (2% ^13^C-enriched glucose solution, black) and WP (wheat porridge with 2% ^13^C-labeled wheat flour, red). **(B)** Concentrations of ^13^C-labeled metabolite isotopologues glucose M6, pyruvate M3, lactate M3, alanine M3, and citrate M2 and protein (derived from glutamate M5, glutamine M5, valine M5 and threonine M4), displayed is the average of 11 study subjects ± standard error of the mean (SEM), statistics: repeated measures (rm) ANOVA. Glc, glucose; Ser, serine; Gly, glycine; Pyr, pyruvate; Lac, lactate; Ala, alanine; Cit, citrate; aKG, α-ketoglutarate; Succ, succinate; Fum, fumarate; Mal, malate; OxAc, oxaloacetate; Glu, glutamate; Gln, glutamine; Thr, threonine; Val, valine; Leu, leucine; Ile, isoleucine; Lys, lysine; Tyr, tyrosine.

To dynamically investigate postprandial metabolism after GLC and WP ingestion, we recorded isotopic enrichment profiles of central carbon metabolites after an oral administration of ^13^C-labeled tracers in a time-resolved manner. The major wheat component is starch which is hydrolyzed into glucose monomers making subsequent metabolism and enrichment patterns in general identical to the GLC intervention (Figure 2A, black circles). However, in addition to starch, wheat flour also contains protein (gluten) and this ^13^C-labeled flour protein is hydrolyzed to free fully ^13^C-enriched amino acids (Figure 2A, red circles).

After consumption of WP, the starch present in wheat flour has to undergo digestion and hydrolysis before glucose monomers can be transferred into the circulation, and other factors, including delayed gastric emptying might also impact on this. Hence, we expected a slight delay in the appearance of glucose M6 in plasma as compared to GLC. However, the observed delay in glucose M6 appearance was only minor, more pronounced in the declining phase and did not reach statistical significance. Thus, gastric emptying was not delayed after WP and the cooked wheat starch underwent rapid hydrolyzation.

In contrast, the profiles of food-derived pyruvate M3, lactate M3, and alanine M3 were significantly altered in WP compared to GLC (Figure 2B). While we observed a slight delay in the increasing branch of pyruvate M3 after WP intake, the concentration of lactate M3 increased considerably steeper and declined faster in GLC. The maximum concentration of 8.3 μM was reached after 90 min in GLC, while the maximum labeled lactate concentration in WP was lower (6.3 μM) and deferred (after 120 min). For alanine M3, a similar pattern was observed and the declining phase of alanine M3 was delayed in WP compared to GLC. Of note, both metabolites, lactate and alanine, are derived from the same precursor pyruvate (Figure 2A) and accumulate to (relatively) high systemic concentrations [lac ~ 2 mM, ala ~ 0.4 mM; www.hmdb.ca (18)]. Citrate M2 showed a similar trend toward a delay in the declining phase, however, without reaching statistical significance. In summary, the quantitative isotopologue data of glucose and its downstream metabolites (Figure 2B) suggested that the intake of GLC induced a faster response reaching higher maximal isotopologue concentrations as compared to WP intake, where the profiles were deferred and damped. Of note, due to the low amount of labeling introduced (~2%), we were unable to detect ^13^C-enrichment in the TCA cycle metabolites downstream of citrate and other metabolic pathways of lower activity.

Considering the nutrient composition of the two interventions, the intake of GLC predominantly causes a dysregulation of systemic glucose concentrations, whereas the intake of wheat flour based products increases the availability of a complex mixture of nutrients including glucose, dietary amino acids, lipids, vitamins, etc. that, once absorbed, can enter central carbon metabolism at various metabolic sites (19, 20). As a consequence, WP intake would result in a situation of excessive nutrient availability compared to the GLC situation, and this could be one of the reasons for the dampened metabolic conversion of exogenous starch-derived glucose.

**Figure 3:**
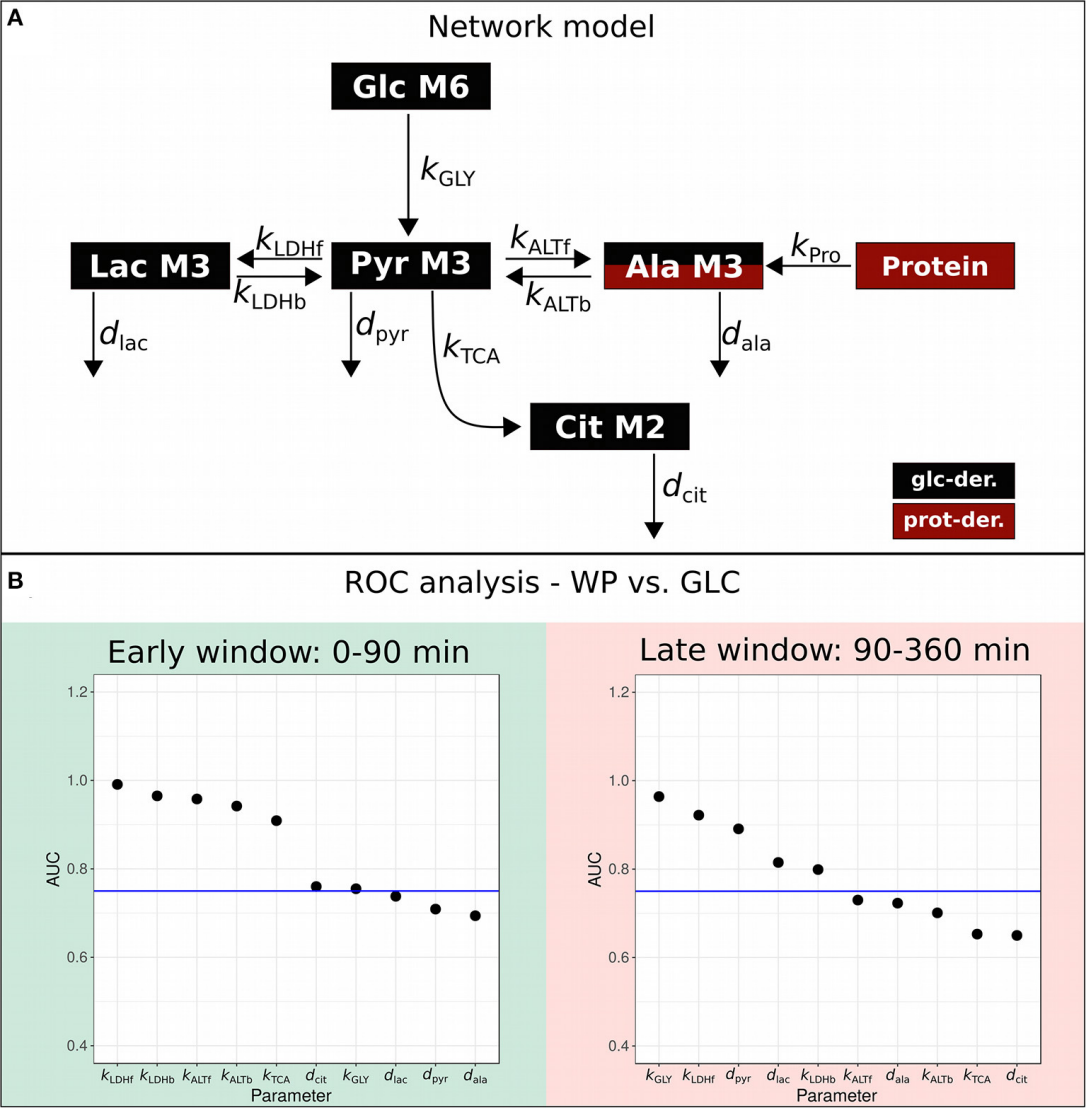
Metabolic network model. **(A)** Metabolic network model. **(B)** ROC-AUC analysis to determine the most differentially regulated parameters, i.e., *k*-values between WP and GLC in the early (0–90 min) and late (90–360 min) postprandial phase; ROC-AUC above 0.75 is considered strongly regulated (blue horizontal line).

**Figure 4:**
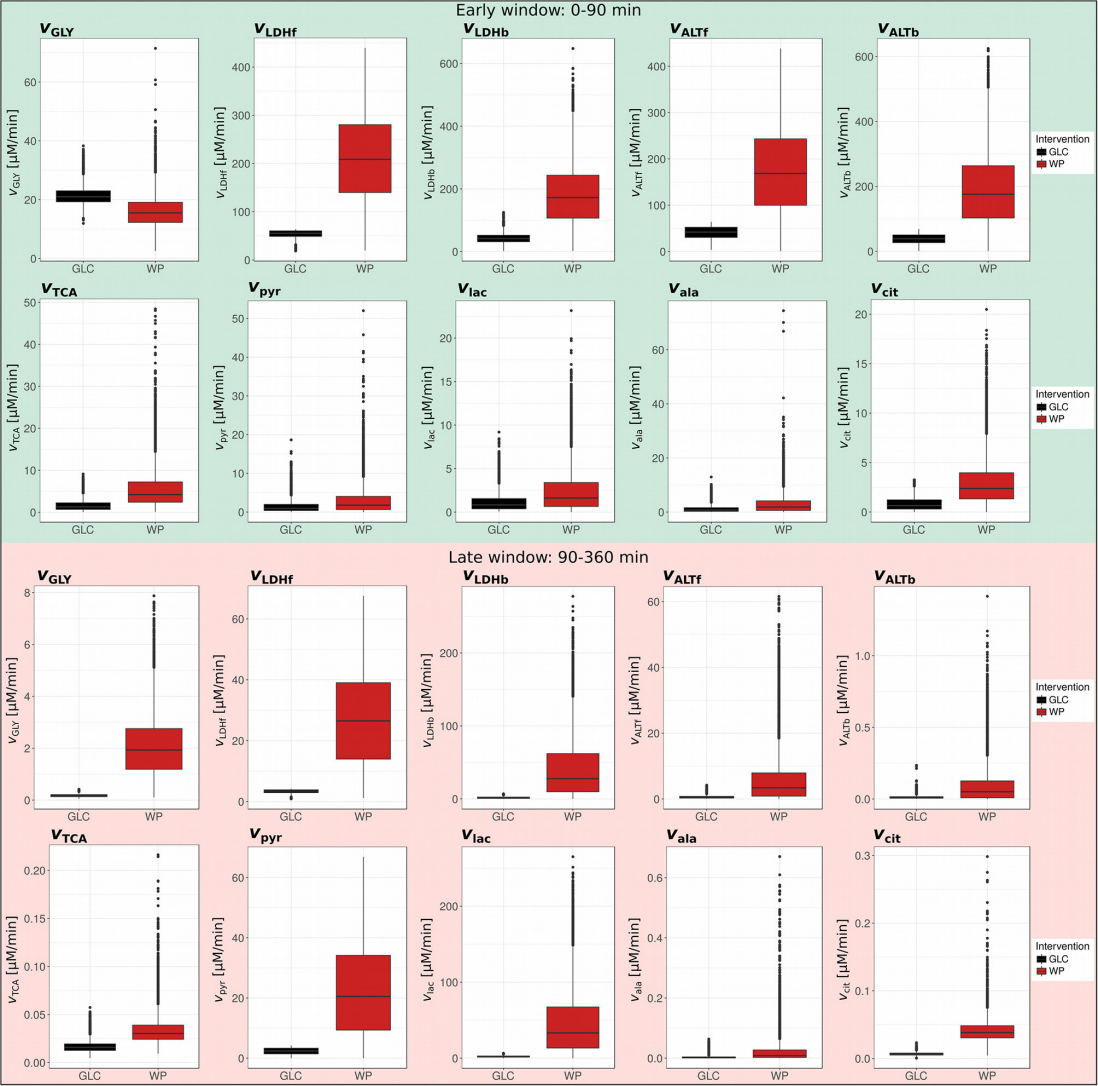
Postprandial metabolic fluxes in the early and late window. Boxplots of simulated postprandial metabolic fluxes in GLC and WP obtained for the early (0–90 min) and late (90–360 min) postprandial phase (GLC, black; WP, red; *n* = 10,000).

To quantitatively compare the dynamic postprandial fluxes of the two interventions, we constructed a kinetic ODE model by taking into account the major glucose-metabolizing pathways, namely glycolysis and TCA cycle (Figure 3A), for both of which the atom transitions are well-established. We applied the quantitative isotopologue data (Figure 2B) to determine the parameters of our metabolic network model presented in Figure 3A (specifically *k*_GLY_, *k*_LDHf_, *k*_LDHb_, *k*_ALTf_, *k*_ALTb_, *k*_TCA_, *d*_pyr_, *d*_lac_, *d*_ala_, and *d*_cit_). The postprandial phase was divided in the absorptive (early window: 0–90 min) and the post-absorptive phase (late window: 90–360 min) and parameters were estimated separately. A logistic regression model was applied on the network model parameters to identify intervention-induced differences in the scope of this model by ROC-AUC analysis (Figure 3B; Supplementary Table S7). The corresponding metabolic fluxes were subsequently derived from the obtained kinetic rates, i.e., *k*-values (Supplementary Table S8; Figure 4). Technical details of the modeling approach are presented in Section Metabolic Network Model.

We first compared our glycolytic flux (*v*_GLY_) to existing data to ensure that the obtained values are physiologically meaningful (Figure 4, early window, green). This parameter can best be compared to the appearance of exogenous glucose (RaE) derived from the dual label technique (21). Importantly, our *v*_GLY_ parameter represents appearance of exogenous glucose and further conversion into pyruvate, thus *v*_GLY_ is a subset of RaE and is expected to be lower. We compared our results with two studies investigating the postprandial metabolism of wheat intake (22, 23), and found that our data was in the same order of magnitude, but slightly lower (~10–40% of reported RaE; Supplementary Table S9). With this, we concluded that our approach is suitable to generate meaningful postprandial flux estimations.

### Increased Metabolite Turn-Over After WP Consumption

ROC-AUC analysis revealed that *k*_LDHf/b_, *k*_ALTf/b_, *k*_TCA_, *d*_cit_, and *k*_GLY_ were differentially regulated in the early window, while *k*_GLY_, *k*_LDHf/b_, *d*_pyr_, and *d*_lac_ were changed in the late window (Figure 3B; Supplementary Table S7). We observed more distinct differences in ROC-AUC in the early phase than in the later phase (Figure 3B) and this trend was sustained in the resulting metabolic fluxes (Figure 4; Supplementary Table S10). In the early window, the glycolytic flux *v*_GLY_ was higher in GLC (21.3 μM/min) than in WP (16.0 μM/min), however, the majority of the other fluxes, including *v*_LDHf/b_, *v*_ALTf/b_, *v*_TCA_ and *v*_cit_, were increased after WP intake (Figure 4, upper panel). Conversion of food-derived pyruvate into citrate was mainly observed after WP intake in the early phase (5.6 μM/min). In the late window, most fluxes were found to be low in magnitude compared to the early window and the intervention induced flux changes were mainly found in the glucose—pyruvate—lactate axis of the metabolic network model (Figure 4, lower panel). We further observed a carbohydrate dependent switch of the glycolytic contribution in the late phase, where *v*_GLY_ was suddenly higher in WP (2.07 μM/min) than in GLC (0.18 μM/min). Other fluxes, including *v*_LDHf/b_, *v*_ALTf/b_, *v*_TCA_ and *v*_cit_ were lower in magnitude, but still higher in WP (Figure 4, lower panel). We further observed that the outgoing fluxes *v*_pyr_ (Early: GLC—1.46 μM/min, WP—3.06 μM/min, late: GLC—2.34 μM/min, and WP—22.6 μM/min) and *v*_lac_ (Early: GLC—1.13 μM/min, WP—2.45 μM/min, late: GLC—2.16 μM/min, and WP—46.42 μM/min) increased substantially in the late window, especially after WP intake.

In summary, metabolic fluxes throughout the postprandial window were higher after WP consumption, indicating an overflow of nutrients. The intervention-induced flux adaptations finally resulted in overall similar glucose levels (Figure 1), thus, these underlying flux adjustments were needed to keep glucose levels in the physiological range. Furthermore, we found more distinct flux changes in the early compared to the later window. This might not be surprising as in the absorptive phase, the system is challenged with two substantially different interventions, GLC and WP, and has to react to the respective intervention in a tailored fashion. In contrast, in the late, post-absorptive phase, the system and the metabolic fluxes return to equilibrium, which is less dependent on the consumed intervention.

### Role of Circulating Lactate and Alanine in the Postprandial Phase

**Figure 5:**
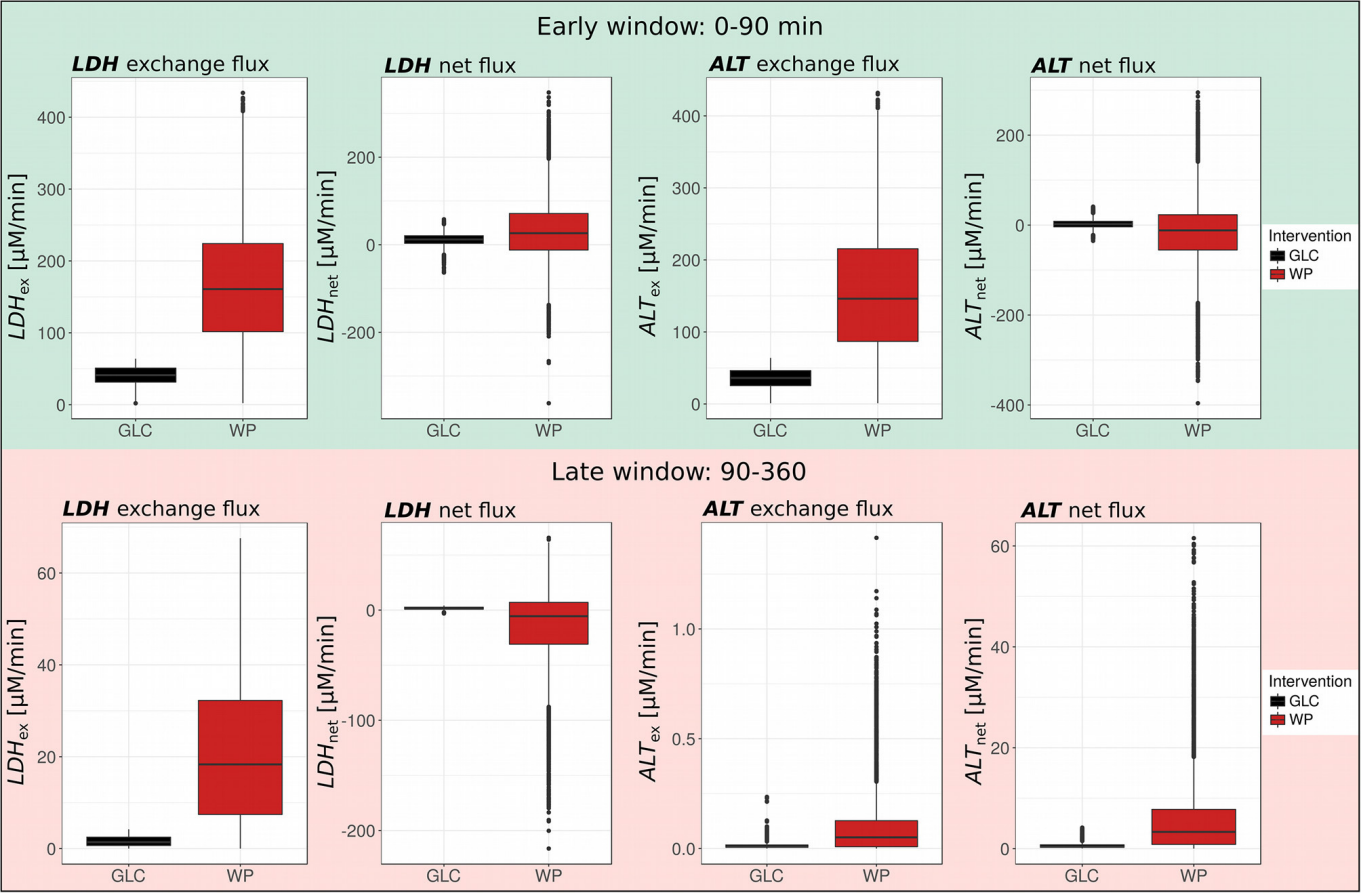
LDH and ALT exchange and net fluxes. Boxplots of LDH and ALT exchange and net fluxes in GLC and WP obtained for the early (0–90 min) and late (90–360 min) postprandial phase (GLC, black; WP, red; *n* = 10,000).

We found a substantial upregulation of the exchange flux of pyruvate and lactate (*LDH*_ex_) both, in the early and the late window after WP intake (Early: WP—166.9 μM/min, GLC—40.7 μM/min; Late: WP—21.2 μM/min, and GLC—1.63 μM/min; Figure 5, left) pointing toward a higher LDH activity in WP. Interestingly, the net direction of the LDH flux (*LDH*_net_) was dependent on the carbohydrate source and the time window. In GLC, we observed net production of lactate during the entire postprandial window (Early: 11.3 μM/l, Late: 1.63 μM/l). In contrast, after WP intake, we observed net production of lactate in the early window (31.2 μM/l) and net production of pyruvate in the late window (14.8 μM/l).

Similar to the LDH exchange flux, the ALT exchange flux of pyruvate and alanine (*ALT*_ex_) was increased in WP in both postprandial windows, however, the exchange flux was much higher in the early (GLC—35.5 μM/min, WP—155.9 μM/l) than in the late window (GLC—0.015 μM/min, WP—0.100 μM/l) (Figure 5, right). While the net ALT flux (*ALT*_net_) was directed toward alanine production in GLC (Early: 2.2 μM/min, Late: 0.69 μM/min), in WP, we observed net pyruvate production from alanine in the early window (16.6 μM/min). This observation was likely driven by the entry of protein-derived alanine, which was only available after WP intake. The direction of the net ALT flux switched in the late window toward net alanine production from pyruvate (6.17 μM) and net flux was always higher in WP compared to GLC. Interestingly, the LDH and ALT exchange fluxes were in a similar magnitude in the early window, however, in the later window and independent of the intervention, the LDH exchange flux was 100–200 fold higher compared to the ALT exchange flux, highlighting the importance of the LDH reaction especially in the late postprandial setting.

### Gluten-Derived Serine and Glycine Strongly Contribute to the Postprandial Plasma Pool

**Figure 6:**
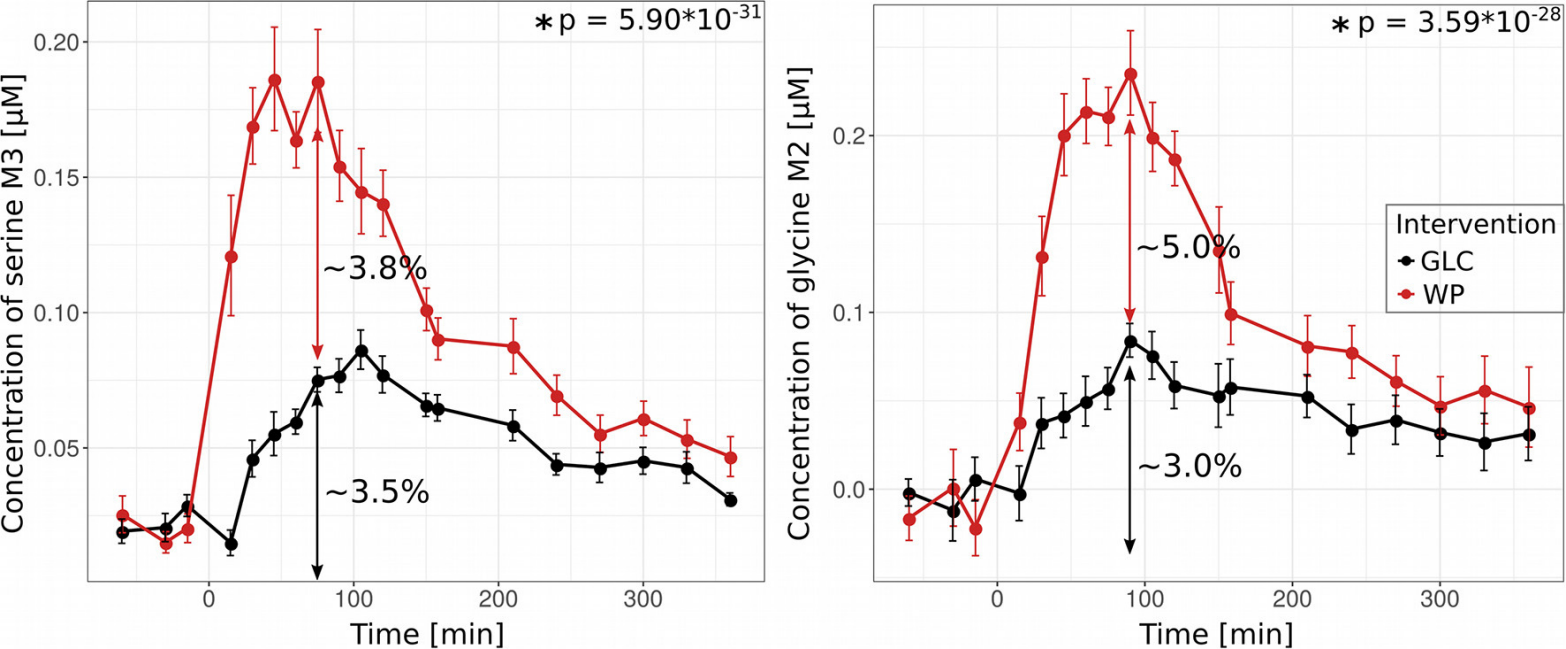
Protein-derived serine and glycine contribute to the plasma pool. Concentrations of ^13^C-labeled isotopologues serine M3 and glycine M2 after glucose (GLC, black) and wheat porridge (WP, red) intake; displayed is the average of eleven subjects ± standard error of the mean (SEM), statistics: rmANOVA.

We next studied how starch/glucose and gluten contribute to the synthesis of the amino acids serine and glycine. Both amino acids play key roles in folate mediated one carbon metabolism and serine is a major donor for methyl groups needed for methylation reactions such as DNA as well as histone methylation and thymidylate synthesis (24). Serine and glycine can either be *de novo* synthesized from glucose or directly be derived from protein hydrolysis, however, both metabolic routes would result in identical enrichment patterns, i.e., fully labeled glycine (M2) and serine (M3) and can thus not be easily distinguished (Figure 2A). To reveal the contributions of both sources, we first quantified the amount of *de novo* synthesized serine and glycine in the GLC situation at maximum (ser: 75 min, gly: 90 min) and found that 3.5% of the plasma serine pool and 3% of the glycine pool were derived from glucose. Because in this intervention, there is no ^13^C contribution from protein hydrolysis, we roughly estimated this contribution by the difference between both interventions (Figure 6). We revealed that 3.8% of the serine and 5% of the glycine pool were derived from protein hydrolysis of the wheat protein (Figure 6). These results demonstrate that both, *de novo* synthesis and protein hydrolysis contribute equally to the respective amino acid pools in plasma. Similar results were also found for the wheat protein-derived amino acids glutamine and glutamate (Supplementary Figure S1). However, in case of alanine, we did not observe any excess alanine M3 after WP consumption (Figure 2B) although our modeling approach determined a significant contribution of protein-derived alanine to its plasma pool (Figures 4, 5). This discrepancy can in part be explained by the lower amount of alanine in gluten protein [Ala—270 μmol/g; Ser—440 μmol/g, Gly—429 μmol/g (20)], which is diluted in a higher baseline plasma pool size of alanine (Ala—225 μM; Ser—120 μM, Gly—140 μM, Supplementary Table S4).

### Appearance of ^13^C-Labeled 3- and 2-Hydroxybutyrate After Intake of WP

**Figure 7:**
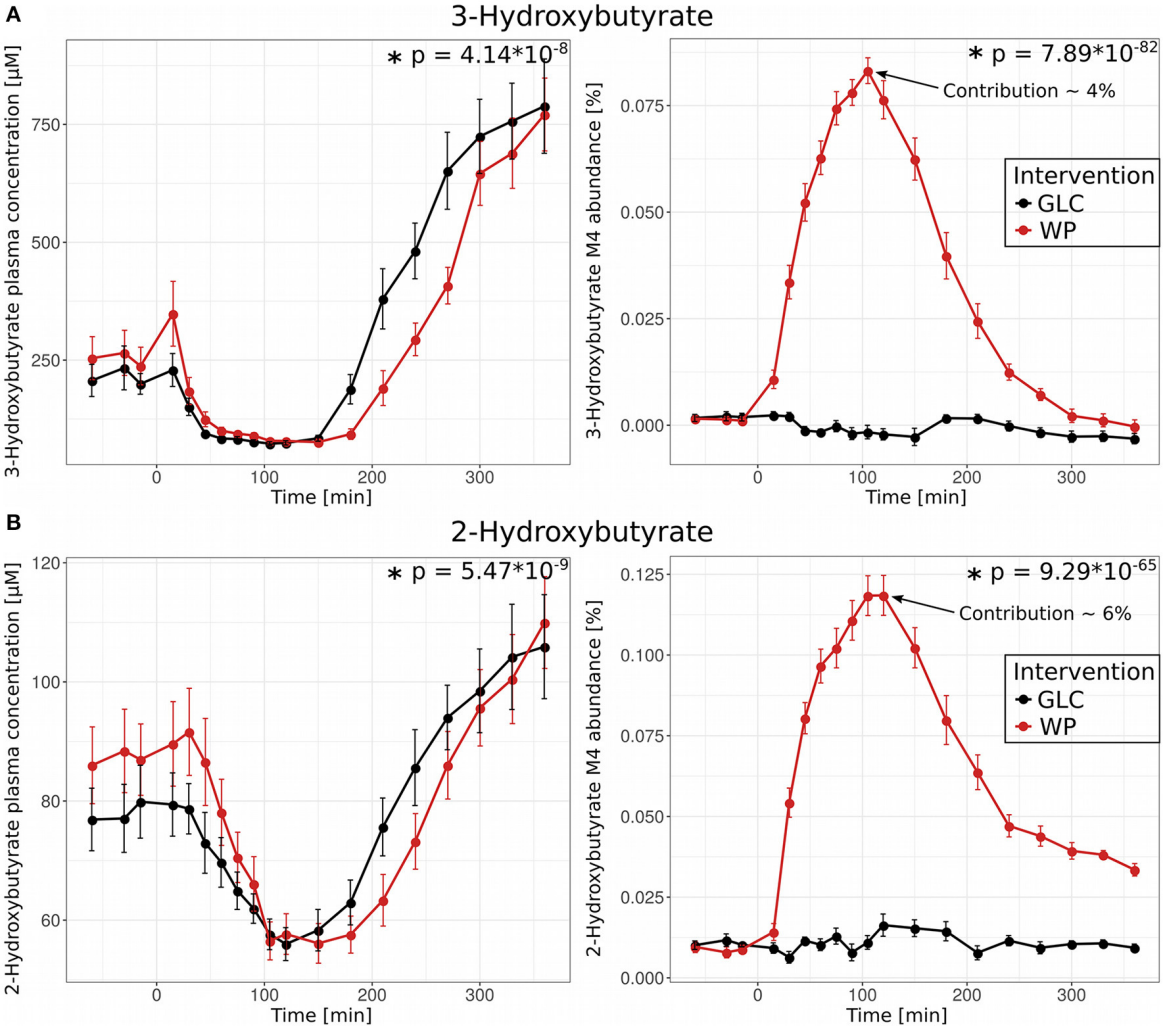
Role of 3- and 2-Hydroxybutyrate in the postprandial phase. **(A)** 3-Hydroxybutyrate and **(B)** 2-Hydroxybutyrate plasma concentrations (left panel) and M4 isotopologue abundances (right panel) and resulting contribution to the total plasma pool 105 min after glucose (GLC, black) and wheat porridge (WP, red) intake; displayed is the average of eleven subjects ± standard error of the mean (SEM), statistics: rmANOVA.

3-hydroxybutyrate (ß-hydroxybutyrate, 3HB) is a ketone-body and fuels significant parts of energy metabolism under fasting conditions. During fasting, plasma levels can reach concentrations up to 2 mM; 3HB is mainly synthesized by the liver from free fatty acids originating from deposited lipids in adipose tissues (25). Although similar in structure, 2-hydroxybutyrate (α-hydroxybutyrate, 2HB) is not known to be involved in lipid catabolism and is not recognized as a significant energy source. 2HB is synthesized from α-ketobutyrate, which is formed during threonine catabolism or methionine/cysteine metabolism/transsulfuration. Increased plasma levels of 2HB have been associated with oxidative stress conditions, type 2 diabetes mellitus (T2DM) and prolonged fasting (26–28). As expected, shortly after food intake, the plasma concentration of 3HB dropped rapidly but increased strongly in the late postprandial phase (Figure 7, upper left panel). This behavior is well-known for the ketone body 3HB as its synthesis is dependent on β-oxidation, which is only active in the absence of insulin (29). Surprisingly, we observed a similar postprandial pattern for 2HB (Figure 7, lower left panel), indicating that 2HB and insulin levels might be linked in a similar fashion. We hypothesize that the delayed increase of plasma concentrations in WP observed for 3HB and 2HB is a direct consequence of insulin remaining longer in the circulation (Figure 1, right panel). The observed correlation between 2HB and insulin levels is not yet fully understood and warrants further investigation.

We further observed substantial amounts of fully labeled (M4) 2HB and 3HB after intake of WP, but not after GLC intake. These M4 fractions reached their maximum 100 min post food intake in WP, a time point at which overall 2- and 3-HB concentrations were at minimum with contributions of ~6% and ~4% to the total plasma pool, respectively (Figure 7, right panel). These curves mirror the profiles of the fully labeled essential amino acids, e.g., valine M5 (Supplementary Figure S2) indicating that the ^13^C-labeled wheat flour must be the source of M4 2HB and M4 3HB. *De novo* synthesis of 3HB from acetyl-CoA can be ruled out as 3HB would in this case be labeled at two carbon atoms (M2) instead of four (M4). It has been shown before that 3HB is present in wheat flour in small quantities (30), further strengthening our hypothesis that 3-hydroxybutyrate was present in the supplemented ^13^C-labeled wheat flour and appeared in the circulation after consumption of the WP, but not in GLC. For 2HB, Lohaus et al. (30) reported that it is not present in wheat flour, however, the kinetics closely mirror those of 3HB. As a second option, 2HB M4 could also be synthesized from threonine *via* threonine deaminase and 2-ketobutyrate, but these open questions remain to be addressed in future studies.

## Conclusion

**Figure 8:**
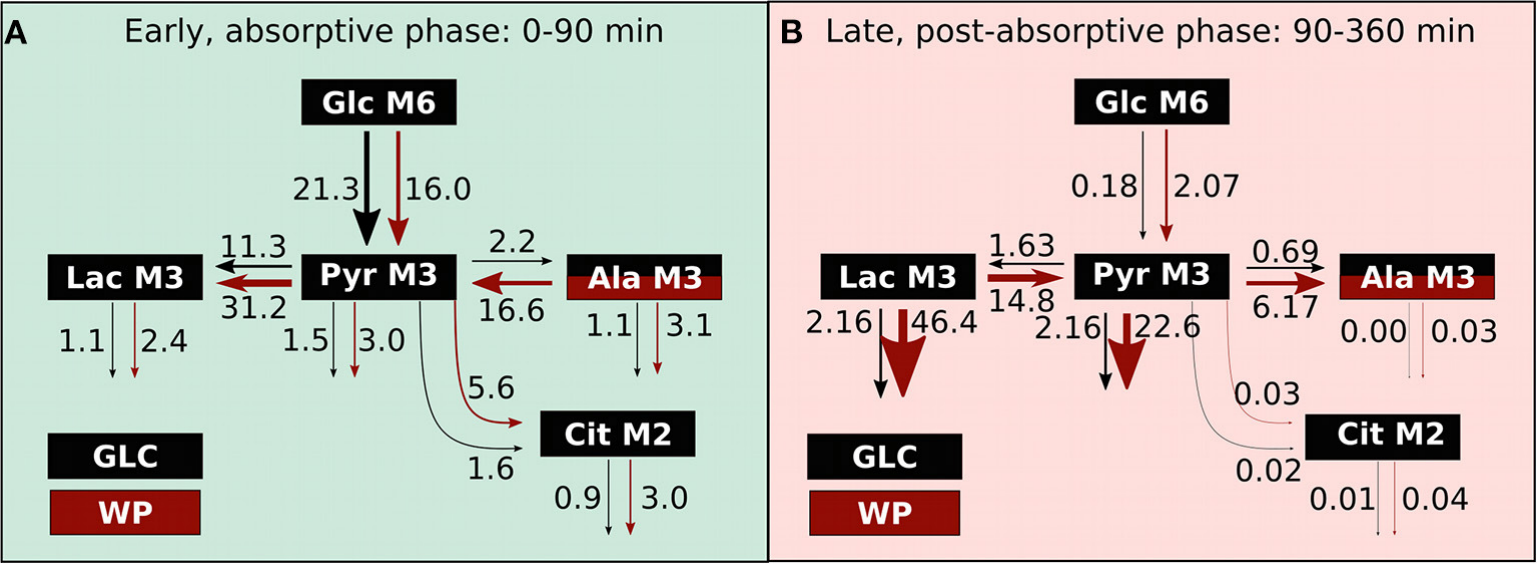
Metabolic fluxes alterations upon GLC and WP intake. Overview of the metabolic flux changes (μM/min) in GLC (black) and WP (red) intervention in the **(A)** early and **(B)** late postprandial window.

The present study was designed to compare postprandial *in vivo* metabolism after glucose consumption with the intake of a wheat-based porridge in human subjects. We separated the entire postprandial phase into two windows, the absorptive (0–90min) and the post-absorptive phase (90–360 min), as underlying metabolic fluxes are adapted in a time and intervention dependent manner (Figure 8). After WP intake, the early, absorptive phase was characterized by an overall increase in metabolic fluxes, with the glycolytic flux being the only exception. In our view, two factors predominantly contributed to this phenotype, namely (I) the trend observed toward a delay in glucose appearance and (II) the availability of protein-derived alanine being converted into pyruvate. LDH and ALT exchange fluxes were four-fold increased in WP indicating that nutrient overflow was coped with at the sites of lactate and alanine. While only a small fraction of food-derived pyruvate was entering the TCA cycle, especially after WP intake, the largest fraction of food-derived metabolites was converted into lactate. In the late, post-absorptive phase, LDH and ALT fluxes changed net directions resulting in conversion of lactate into pyruvate and further into alanine after WP consumption. Moreover, substantial amounts of lactate and pyruvate were removed from the plasma pool pointing toward a redirection of predominantly pyruvate and lactate from the blood compartment into tissues, i.e., liver and muscle where they act as substrates for energy production, glycogen synthesis, and gluconeogenesis (31–34). Importantly, in contrast to WP, GLC intake did not alter net LDH and ALT flux directions, instead we observed lactate and alanine net production from pyruvate over the entire postprandial window and flux magnitudes were always significantly lower. However, overall plasma pool sizes including glucose (Figure 1), pyruvate, lactate and alanine (Supplementary Figure S3) were not altered between GLC and WP. Our data clearly confirm that metabolic homeostasis of systemic metabolites is tightly and robustly regulated (1, 2, 32). To maintain metabolite concentrations in physiologically acceptable ranges, the organism relies on quick and significant adjustments of producing and consuming metabolic fluxes in a nutrient dependent manner. Overall, we observed increased credible intervals after WP compared to GLC consumption (Figure 4; Supplementary Table S10), indicating that the variability in the metabolic response is higher after intake of a complex mixture of nutrients. Mixed meal nutritional studies might thus require increased numbers of participants to cope with this inhomogeneity.

Our findings indicate that lactate, pyruvate and, to a lesser extent, alanine serve as systemic metabolic buffers in the postprandial setting to cope with the nutrient load after food intake. Systemic lactate levels increased up to three-fold and pyruvate levels up to two-fold, while the maximal increase observed for alanine and glucose was more limited (Ala: 1.3×, Supplementary Figure S4; Glc: 1.5×, Figure 1). Especially lactate has a higher physiological flexibility in terms of systemic concentrations and we thus suggest that lactate takes a key role in postprandial metabolite homeostasis.

Our study further confirmed that dietary protein-derived amino acids alanine, serine and glycine contribute to their respective plasma metabolite pools and unveiled an interesting correlation between 2HB and 3HB levels. The appearance of uniformly labeled M4 2- and 3HB indicates that both metabolites are present in wheat flour and are taken up into the circulation.

In conclusion, we demonstrated that *in vivo* metabolic fluxes of central carbon metabolism were regulated differentially in a nutrient dependent manner, while plasma metabolite concentrations were only modestly affected. Our findings thus underscore that metabolite levels do not tell the whole story and that it is essential to consider metabolic fluxes in order to extend our knowledge on the regulatory mechanisms in postprandial metabolism. In the future, mixed meal nutritional interventions combined with metabolic flux determination hold promise as highly accurate and early markers for the detection of disease-related changes in metabolism.

## Data Availability Statement

The datasets presented in this study are uploaded in an online repository (https://doi.org/10.24355/dbbs.084-202109291537-0) and can also be found in the article/Supplementary Material.

## Ethics Statement

The studies involving human participants were reviewed and approved by Medical Ethics Committee Brabant, Tilburg, NL. The patients/participants provided their written informed consent to participate in this study.

## Author Contributions

DJ and KH conceived the experiment. HB was involved in designing the study. LS performed metabolite extraction, GC-MS measurements, data and statistical analysis, and wrote the manuscript. GZ, C-AD, and MM-H performed metabolic modeling. GZ, HB, DJ, and KH revised the manuscript. All authors read and approved the content of the final manuscript.

## Funding

LS received funding from Unilever. GZ was supported by the Helmholtz Alliance Aging and Metabolic Programming, AMPro.

## Conflict of Interest

HB and DJ are employed by the company Unilever, LS received funding from Unilever. The remaining authors declare that the research was conducted in the absence of any commercial or financial relationships that could be construed as a potential conflict of interest. The authors declare that this study received funding from Unilever. The funder was involved in the study design, data collection, decision to publish, and manuscript preparation.

## Publisher's Note

All claims expressed in this article are solely those of the authors and do not necessarily represent those of their affiliated organizations, or those of the publisher, the editors and the reviewers. Any product that may be evaluated in this article, or claim that may be made by its manufacturer, is not guaranteed or endorsed by the publisher.

## References

[1] Broer S Broer A. Amino acid homeostasis and signalling in mammalian cells and organisms. Biochem J. (2017) 474:1935–63. 10.1042/BCJ20160822 28546457 PMC5444488

[2] Konig M Bulik S Holzhutter HG. Quantifying the contribution of the liver to glucose homeostasis: a detailed kinetic model of human hepatic glucose metabolism. PLoS Comput Biol. (2012) 8:e1002577. 10.1371/journal.pcbi.1002577 22761565 PMC3383054

[3] Montagner A Polizzi A Fouche E Ducheix S Lippi Y Lasserre F. Liver PPARalpha is crucial for whole-body fatty acid homeostasis and is protective against NAFLD. Gut. (2016) 65:1202–14. 10.1136/gutjnl-2015-310798 26838599 PMC4941147

[4] Slupsky CM He X Hernell O Andersson Y Rudolph C Lonnerdal B. Postprandial metabolic response of breast-fed infants and infants fed lactose-free vs regular infant formula: a randomized controlled trial. Sci Rep. (2017) 7:3640. 10.1038/s41598-017-03975-4 28623320 PMC5473881

[5] Schmedes M Balderas C Aadland EK Jacques H Lavigne C Graff IE. The effect of lean-seafood and non-seafood diets on fasting and postprandial serum metabolites and lipid species: results from a randomized crossover intervention study in healthy adults. Nutrients. (2018) 10:598. 10.3390/nu10050598 29751643 PMC5986478

[6] Khakimov B Jespersen B Engelsen S. Comprehensive and comparative metabolomic profiling of wheat, barley, oat and rye using gas chromatography-mass spectrometry and advanced chemometrics. Foods. (2014) 3:569–85. 10.3390/foods3040569 28234338 PMC5302243

[7] Jang C Chen L Rabinowitz JD. Metabolomics and isotope tracing. Cell. (2018) 173:822–37. 10.1016/j.cell.2018.03.055 29727671 PMC6034115

[8] Baxter CJ Liu JL Fernie AR Sweetlove LJ. Determination of metabolic fluxes in a non-steady-state system. Phytochemistry. (2007) 68:2313–9. 10.1016/j.phytochem.2007.04.026 17582446

[9] Priebe MG Wachters-Hagedoorn RE Heimweg JA Small A Preston T Elzinga H. An explorative study of *in vivo* digestive starch characteristics and postprandial glucose kinetics of wholemeal wheat bread. Eur J Nutr. (2008) 47:417–23. 10.1007/s00394-008-0743-6 18853232

[10] Peronnet F Meynier A Sauvinet V Normand S Bourdon E Mignault D. Plasma glucose kinetics and response of insulin and GIP following a cereal breakfast in female subjects: effect of starch digestibility. Eur J Clin Nutr. (2015) 69:740–5. 10.1038/ejcn.2015.50 25852025 PMC4458892

[11] Eelderink C Noort MWJ Sozer N Koehorst M Holst JJ Deacon CF. Difference in postprandial GLP-1 response despite similar glucose kinetics after consumption of wheat breads with different particle size in healthy men. Eur J Nutr. (2017) 56:1063–76. 10.1007/s00394-016-1156-6 26857762 PMC5346412

[12] Quek LE Krycer JR Ohno S Yugi K Fazakerley DJ Scalzo R. Dynamic (13)C flux analysis captures the reorganization of adipocyte glucose metabolism in response to insulin. iScience. (2020) 23:100855. 10.1016/j.isci.2020.100855 32058966 PMC7005519

[13] Binsl TW De Graaf AA Venema K Heringa J Maathuis A De Waard P. Measuring non-steady-state metabolic fluxes in starch-converting faecal microbiota *in vitro*. Benef Microbes. (2010) 1:391–405. 10.3920/BM2010.0038 21831778

[14] Kramer L Jager C Trezzi JP Jacobs DM Hiller K. Quantification of stable isotope traces close to natural enrichment in human plasma metabolites using gas chromatography-mass spectrometry. Metabolites. (2018) 8:15. 10.3390/metabo8010015 29443915 PMC5876004

[15] Schlicker L Boers HM Dudek CA Zhao G Barua A Trezzi JP. Postprandial metabolic effects of fiber mixes revealed by *in vivo* stable isotope labeling in humans. Metabolites. (2019) 9:91. 10.3390/metabo9050091 31067731 PMC6571904

[16] Hiller K Hangebrauk J Jager C Spura J Schreiber K Schomburg D. MetaboliteDetector: comprehensive analysis tool for targeted and nontargeted GC/MS based metabolome analysis. Anal Chem. (2009) 81:3429–39. 10.1021/ac802689c 19358599

[17] Jennings ME Matthews DE. Determination of complex isotopomer patterns in isotopically labeled compounds by mass spectrometry. Anal Chem. (2005) 77:6435–44. 10.1021/ac0509354 16194110 PMC2268020

[18] Wishart DS Feunang YD Marcu A Guo AC Liang K Vazquez-Fresno R. HMDB 4.0: the human metabolome database for 2018. Nucleic Acids Res. (2018) 46:D608–D17. 10.1093/nar/gkx1089 29140435 PMC5753273

[19] Shewry PR Hey SJ. The contribution of wheat to human diet and health. Food Energy Secur. (2015) 4:178–202. 10.1002/fes3.64 27610232 PMC4998136

[20] Rombouts I Lamberts L Celus I Lagrain B Brijs K Delcour JA. Wheat gluten amino acid composition analysis by high-performance anion-exchange chromatography with integrated pulsed amperometric detection. J Chromatogr A. (2009) 1216:5557–62. 10.1016/j.chroma.2009.05.066 19523641

[21] Boers HM Alssema M Mela DJ Peters HPF Vonk RJ Priebe MG. The rate of glucose appearance is related to postprandial glucose and insulin responses in adults: a systematic review and meta-analysis of stable isotope studies. J Nutr. (2019) 149:1896–903. 10.1093/jn/nxz150 31291451

[22] Ostman JR Mullner E Eriksson J Kristinsson H Gustafsson J Witthoft C. Glucose appearance rate rather than the blood glucose concentrations explains differences in postprandial insulin responses between wholemeal rye and refined wheat breads-results from a cross-over meal study. Mol Nutr Food Res. (2019) 63:e1800959. 10.1002/mnfr.201800959 30636184

[23] Boers HM van Dijk TH Hiemstra H Hoogenraad AR Mela DJ Peters HPF. Effect of fibre additions to flatbread flour mixes on glucose kinetics: a randomised controlled trial. Br J Nutr. (2017) 118:777–87. 10.1017/S0007114517002781 29110741

[24] Lan X Field MS Stover PJ. Cell cycle regulation of folate-mediated one-carbon metabolism. Wiley Interdiscip Rev Syst Biol Med. (2018) 10:e1426. 10.1002/wsbm.1426 29889360 PMC11875019

[25] Newman JC Verdin E. Ketone bodies as signaling metabolites. Trends Endocrinol Metab. (2014) 25:42–52. 10.1016/j.tem.2013.09.002 24140022 PMC4176946

[26] Varvel SA Pottala JV Thiselton DL Caffrey R Dall T Sasinowski M. Serum alpha-hydroxybutyrate (alpha-HB) predicts elevated 1 h glucose levels and early-phase beta-cell dysfunction during OGTT. BMJ Open Diabetes Res Care. (2014) 2:e000038. 10.1136/bmjdrc-2014-000038 25452875 PMC4212560

[27] Rubio-Aliaga I De Roos B Duthie SJ Crosley LK Mayer C Horgan G. Metabolomics of prolonged fasting in humans reveals new catabolic markers. Metabolomics. (2011) 7:375–87. 10.1007/s11306-010-0255-2

[28] Dudzik D Zorawski M Skotnicki M Zarzycki W Garcia A Angulo S. GC-MS based Gestational Diabetes Mellitus longitudinal study: Identification of 2-and 3-hydroxybutyrate as potential prognostic biomarkers. J Pharm Biomed Anal. (2017) 144:90–8. 10.1016/j.jpba.2017.02.056 28314466

[29] Grabacka M Pierzchalska M Dean M Reiss K. Regulation of ketone body metabolism and the role of PPARalpha. Int J Mol Sci. (2016) 17:2093. 10.3390/ijms17122093 27983603 PMC5187893

[30] Lohaus E Bios I Rudiger W. Carboxylic acids in wheat, rye and barley. Z Naturforsch. (1983) 38:524–30. 10.1515/znc-1983-7-805

[31] Radziuk J Pye S. Hepatic glucose uptake, gluconeogenesis and the regulation of glycogen synthesis. Diabetes Metab Res Rev. (2001) 17:250–72. 10.1002/dmrr.217 11544610

[32] Rabinowitz JD Enerback S. Lactate: the ugly duckling of energy metabolism. Nat Metab. (2020) 2:566–71. 10.1038/s42255-020-0243-4 32694798 PMC7983055

[33] Dimitriadis GD Maratou E Kountouri A Board M Lambadiari V. Regulation of postabsorptive and postprandial glucose metabolism by insulin-dependent and insulin-independent mechanisms: an integrative approach. Nutrients. (2021) 13:159. 10.3390/nu13010159 33419065 PMC7825450

[34] Cori CF. The glucose-lactic acid cycle and gluconeogenesis. Curr Top Cell Regul. (1981) 18:377–87. 10.1016/B978-0-12-152818-8.50028-1 7273846

