# Supplementary material for: Systemic Lactate Acts as a Metabolic Buffer in Humans and Prevents Nutrient Overflow in the Postprandial Phase
Source: Front Nutr. 2022 Mar 10;9:785999. doi: 10.3389/fnut.2022.785999 (PMC8961325; doi:10.3389/fnut.2022.785999)
Supplement: Supplementary file 1 [file Data_Sheet_1.zip › Schlicker2022_SupplementalMaterial.docx]

Supplementary Material

# Supplementary Data

**Supplemental method S1:** Inclusion/Exclusion criteria

*Inclusion criteria:*

Eligible subjects must meet all of the following inclusion criteria:

1. Informed consent signed.

2. Healthy Caucasian male.

3. Age at start of the study ≥ 20 and ≤ 50 years.

4. Body mass index (BMI) ≥ 18.0 and ≤ 25.0 kg/m^2^.

5. Apparently healthy: no medical conditions which might affect the study measurements including diabetes type 1 and type 2, gastrointestinal dysfunction, gastrointestinal surgery and inflammatory diseases.

6. Fasting blood glucose value of volunteer is ≥ 3.4 and ≤ 6.1 mmol/liter (i.e. 62-110 mg/dl) at screening.

7. HbA1c ≤ 6.5 % (48 mmol/mol).

8. Having a general practitioner.

9. Agreeing to be informed about medically relevant personal test-results by a physician.

10. Has the ability to lie in a semi-recumbent position for at least 8 hours.

11. Willing to comply to study protocol during study.

12. Accessible veins on arms as determined by examination at screening.

*Exclusion citeria*

Eligible subjects must meet none of the following exclusion criteria:

1. Use of antibiotics within 3 months before Day 1; use of any other medication except paracetamol within 14 days before Day 1.

2. Blood donation in the past 3 months.

3. Reported participation in another nutritional or biomedical trial 3 months before the pre-study examination or during the study.

4. Reported participation in night shift work two weeks prior to pre-study investigation or during the study. Night work is defined as working between midnight and 6.00 AM.

5. Reported intense sporting activities > 10h/w.

6. Not being used to eat breakfast.

7. Consumption of > 21 alcoholic drinks (equally divided over the week) in a typical week.

8. Reported use of any foods, drinks, supplements and herbs enriched with vitamins, within 14 days prior to Day 1 of the study.

9. Reported use of any nicotine containing products in the six months preceding the study and during the study itself.

10. Reported dietary habits: medically prescribed diet, slimming diet, not used to eat 3 meals a day and/or vegetarian.

11. Reported weight loss/gain (>10%) in the last six months before the study.

12. Positive drug screen or alcohol breath test during the screening and/or at Day -1.

13. Clinically relevant abnormalities in clinical chemistry, hemoglobin or positive HIV, HbsAg and/or HepC at screening.

14. Being an employee of Unilever or QPS Netherlands B.V..

15. Dislike, allergy or intolerance to test products or other food products provided during the study.

The investigator QPS Netherlands B.V. has ensured that subjects anonymity is maintained. Documents identifying the subjects are kept by the investigator at strict confidence.

**Supplemental method S2:** ^13^C-enriched wheat flour

The porridge flour was ^13^C-enriched by the substitution of ~2% of total carbohydrates by ^13^C highly-enriched carbohydrates (>97% atom%). The wheat flour originated from seeds cultured in a ^13^CO_2_-enriched atmosphere at Isolife (Wageningen, The Netherlands). To ensure a high resemblance of the labelled wheat flour and the unlabelled version, the seeds of the U[1]^13^C labelled wheat (Triticum aestivum cv Baldus) were of the same pedigree as the unlabeled seeds (German B wheat variety). To ensure that the labelled flour was representative for the other (unlabeled flour), milling of the ^13^C-enriched seeds has been executed at Meneba (Rotterdam, The Netherlands) at the same time with the unlabeled seeds, starting with increasing the moisture content of both kernels to 12.7%. After this, the labeled and unlabeled seed were mixed with a dilution factor of ~ 1:50 and this whole mix was moisturized to a water content of 16.5% which is suitable for milling. The enrichment of the wheat flour was measured on an isotope-ratio mass spectrometry (IRMS) that was specifically prepared for analyses of highly enriched ^13^C-products (ISOTEC Stable Isotope Division, Miamisburg, OH, USA). According to the IRMS analysis, the ^13^C content present in the ^13^C wheat flour sample was determined at 97.1 atom % ^13^C. Before the start of the study, the ratio between the 13C and 12C starch in the mix of ^13^C flour and the “normal wheat” flour was measured in three representative samples with NMR spectroscopy to confirm the homogeneity of the flour mixtures.

Composition of test porridge and glucose drink

| Treatment | Wheat flour/glucose | | | Total carbs (g) | Dietary fibre (g) |
| --- | --- | --- | --- | --- | --- |
|  | ^13^C labelled ^(g)^ | unlabelled (g) | Total (g) |  |  |
| Porridge* | 1.38 | 67.62 | 69 | 50 | 2 |
| Glucose drink | 1.01 | 48.99 | 50 | 50 |  |

*2.00 atom% excess (APE) 13C

# Supplementary Figures and Tables

## Supplementary Figures

**Supplementary Figure 1.** Labeled concentrations of glutamate and glutamine M5 after glucose (GLC – black) and wheat product (WP – red) intake in µM over time and percentage contribution at maximum; average of eleven subjects ± standard error of the mean (SEM), statistics: rmANOVA with subsequent Benjamini-Hochberg correction


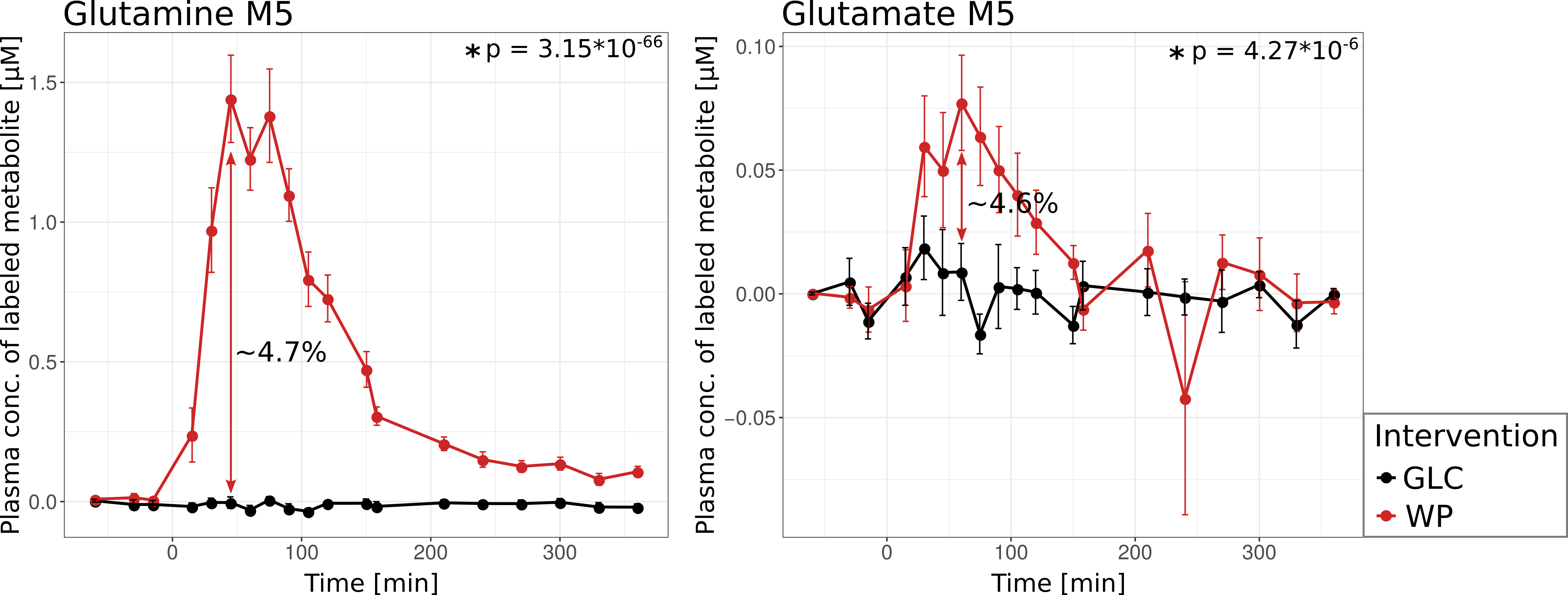


**Supplementary Figure 2.** ^13^C-enrichment profiles of all labeled metabolites categorized by origin (glucose-derived/protein-derived/mixed/unknown) after GLC (black) and WP (red) in % over time; average of eleven subjects ± standard error of the mean (SEM), statistics: rmANOVA with subsequent Benjamini-Hochberg correction





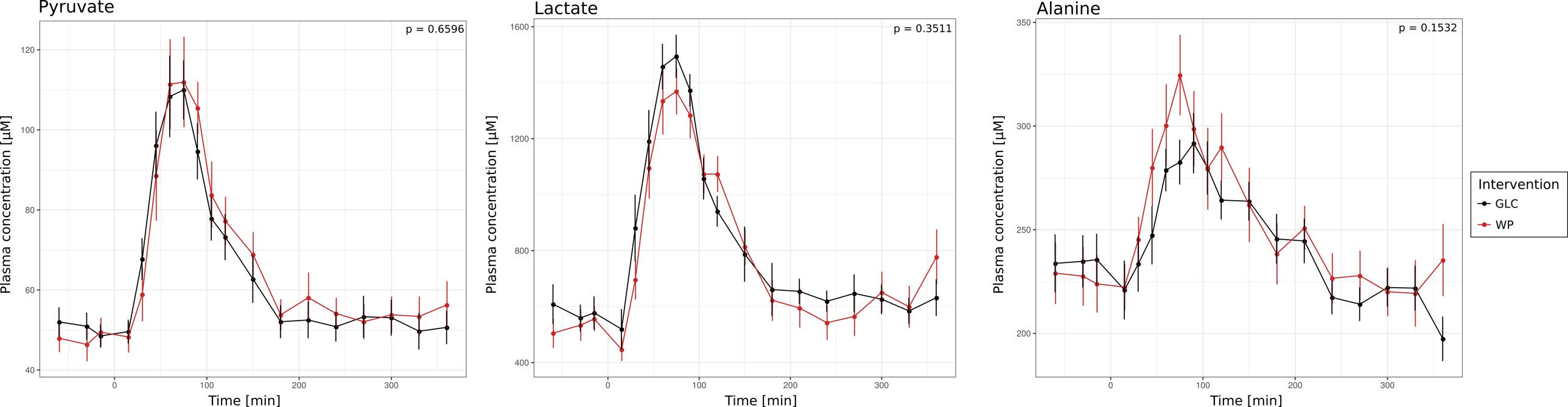
**Supplementary Figure 3.** Plasma concentrations of pyruvate, lactate and alanine after GLC (black) and WP (red) in µM over time; average of eleven subjects ± standard error of the mean (SEM), statistics: rmANOVA with subsequent Benjamini-Hochberg correction

## Supplementary Tables

**Supplementary Table 1.** Mass isotopomer distributions (MIDs) of 11 subjects, all time points (301-319), for both interventions (GLC/WP) (https://doi.org/10.24355/dbbs.084-202109291537-0)

**File:** TableS1_MIDs_GLCWP_OutliersRemoved_MedianOfTriplicates.csv

## Supplementary Table 2. Standards for external calibration from highest (Conc. 1) to lowest (Conc. 6) concentration in µM scaled around commonly observed metabolite concentration observed in blood (taken from www.hmdb.ca)

| **Metabolite** | **c in blood [µM]** | **Conc. 1** | **Conc. 2** | **Conc. 3** | **Conc. 4** | **Conc. 5** | **Conc. 6** |
| --- | --- | --- | --- | --- | --- | --- | --- |
| Pyruvate | 70 | 87.5 | 70 | 56 | 44.8 | 35.84 | 28.672 |
| Lactate | 2000 | 2500 | 2000 | 1600 | 1280 | 1024 | 819.2 |
| 3-Hydroxybutyrate | 100 | 125 | 100 | 80 | 64 | 51.2 | 40.96 |
| 2-Hydroxybutyrate | 60 | 75 | 60 | 48 | 38.4 | 30.72 | 24.576 |
| Citrate | 100 | 125 | 100 | 80 | 64 | 51.2 | 40.96 |
| Malate | 8 | 10 | 8 | 6.4 | 5.12 | 4.096 | 3.2768 |
| Oxalate | 8 | 10 | 8 | 6.4 | 5.12 | 4.096 | 3.2768 |
| Fumarate | 8 | 10 | 8 | 6.4 | 5.12 | 4.096 | 3.2768 |
| Succinate | 20 | 25 | 20 | 16 | 12.8 | 10.24 | 8.192 |
| Alanine | 400 | 500 | 400 | 320 | 256 | 204.8 | 163.84 |
| Serine | 150 | 187.5 | 150 | 120 | 96 | 76.8 | 61.44 |
| Glycine | 250 | 312.5 | 250 | 200 | 160 | 128 | 102.4 |
| Aspartate | 20 | 25 | 20 | 16 | 12.8 | 10.24 | 8.192 |
| Glutamine | 500 | 625 | 500 | 400 | 320 | 256 | 204.8 |
| Glutamate | 80 | 100 | 80 | 64 | 51.2 | 40.96 | 32.768 |
| Lysine | 200 | 250 | 200 | 160 | 128 | 102.4 | 81.92 |
| Leucine | 150 | 187.5 | 150 | 120 | 96 | 76.8 | 61.44 |
| Isoleucine | 80 | 100 | 80 | 64 | 51.2 | 40.96 | 32.768 |
| Valine | 250 | 312.5 | 250 | 200 | 160 | 128 | 102.4 |
| Threonine | 150 | 187.5 | 150 | 120 | 96 | 76.8 | 61.44 |
| Tyrosine | 80 | 100 | 80 | 64 | 51.2 | 40.96 | 32.768 |
| Phenylalanine | 60 | 75 | 60 | 48 | 38.4 | 30.72 | 24.576 |
| Glucose | 5500 | 6875 | 5500 | 4400 | 3520 | 2816 | 2252.8 |

**Supplementary Table 3.** Plasma metabolite concentrations in µM of eleven subjects, for all time points (301-319) and both interventions (GLC/WP) (https://doi.org/10.24355/dbbs.084-202109291537-0)

**File:** TableS3_AbsQuant_GLCWP_OutliersRemoved_MedianOfTriplicates.csv

**Supplementary Table 4.** Plasma metabolite concentrations in µM as average and SE of eleven study subjects

**File:** TableS4_AbsoluteQuantification_AverageSE.xlsx

**Supplementary Table 5.** Scaling factors calculated based on literature values and own data

| **Metabolite** | **Time window** | **Plasma conc. [µM]** | **Tissue conc. [µM]** | **Scaling factor (Tissue/Plasma)** | **References** |
| --- | --- | --- | --- | --- | --- |
| **Lactate** | Early | 1206 | 2757 | 2.29 | [1]–[3]⁠ |
|  | Late | 622.5 | 1817.5 | 2.92 |  |
| **Pyruvate** | Early | 110 | 246.3 | 2.24 | [2],[4],[5]⁠ |
|  | Late | 52.5 | 201.5 | 3.83 |  |
| **Alanine** | Early | 290 | 604 | 2.08 | [6],[7]⁠ |
|  | Late | 233 | 347.7 | 1.49 |  |
| **Citrate** | Early | 110 | 75.25 | 0.68 | [8]–[10]⁠ |
|  | Late | 147.5 | 75.25 | 0.51 |  |

**Supplementary Table 6.** Selection of time point for cutting the early and late windows

| **separating point (min)** | **best cost_early** | **best cost_late** | **best cost_early + best cost_late** | **best cost_whole** | **improvement %**  **(whole-early-late)/whole** |
| --- | --- | --- | --- | --- | --- |
| **75** | 1.0479 | 2.5737 | 3.6216 | 7.1796 | 49.56 |
| **90** | 1.4473 | 2.1959 | 3.6432 | 7.1796 | 49.26 |
| **105** | 2.8225 | 1.4379 | 4.2604 | 7.1796 | 40.66 |
| **120** | 3.7817 | 1.2246 | 5.0063 | 7.1796 | 30.27 |
| **150** | 4.3181 | 0.7871 | 5.1052 | 7.1796 | 28.89 |

**Supplementary Table 7.** Confidence intervals of ROC-AUC analysis for the early (a) and late (b) time window

1. 0-90 min, GLC vs WP

| **Parameter** | **Mean AUC** | **Upper limit** | **Lower limit** | **SD** |
| --- | --- | --- | --- | --- |
| ***k*_LDHf_** | 0.991 | 0.994 | 0.987 | 0.00169 |
| ***k*_LDHb_** | 0.965 | 0.970 | 0.958 | 0.00298 |
| ***k*_ALTf_** | 0.958 | 0.966 | 0.952 | 0.00328 |
| ***k*_ALTb_** | 0.942 | 0.949 | 0.935 | 0.00344 |
| ***k*_TCA_** | 0.909 | 0.917 | 0.902 | 0.00386 |
| ***d*_cit_** | 0.760 | 0.773 | 0.747 | 0.00684 |
| ***k*_GLY_** | 0.755 | 0.768 | 0.740 | 0.00683 |
| ***d*_lac_** | 0.738 | 0.753 | 0.723 | 0.00751 |
| ***d*_pyr_** | 0.709 | 0.722 | 0.697 | 0.0071 |
| ***d*_ala_** | 0.694 | 0.710 | 0.679 | 0.00756 |

1. 90-360 min, GLC vs WP

| **Parameter** | **Mean AUC** | **Upper limit** | **Lower limit** | **SD** |
| --- | --- | --- | --- | --- |
| ***k*_GLY_** | 0.964 | 0.969 | 0.957 | 0.00297 |
| ***k*_LDHf_** | 0.922 | 0.930 | 0.914 | 0.00439 |
| ***d*_pyr_** | 0.891 | 0.902 | 0.881 | 0.00507 |
| ***d*_lac_** | 0.815 | 0.828 | 0.805 | 0.00637 |
| ***k*_LDHb_** | 0.799 | 0.810 | 0.786 | 0.00628 |
| ***k*_ALTf_** | 0.730 | 0.744 | 0.716 | 0.00771 |
| ***d*_ala_** | 0.723 | 0.735 | 0.708 | 0.00718 |
| ***k*_ALTb_** | 0.701 | 0.716 | 0.687 | 0.00779 |
| ***k*_TCA_** | 0.653 | 0.666 | 0.641 | 0.00721 |
| ***d*_cit_** | 0.650 | 0.665 | 0.638 | 0.00713 |

**Supplementary Table 8.** *k* values for all investigated time windows

**File:** TableS8_kvalues_GlcWP_EarlyLate.xlsx

**Supplementary Table 9.** Comparison of our data with literature after harmonizing the units (own data were multiplied by the blood volume assumed to be 5 l [11]⁠)

| Parameter | Intervention | Available Carbohydrates [g] | Range [µmol/min] | Reference |
| --- | --- | --- | --- | --- |
| *v*_GLY_ | Wheat porridge | 50 | 80-105 | Own data |
| RaE | Wheat bread | 50 | 1042-2406 | Östmann *et al.*[12]⁠ |
| RaE | Wheat bread | 69.5 | 218-1300 | Boers *et al.* [13]⁠ |

**Supplementary Table 10.** Summary of postprandial fluxes for early (0-90 min; green) and late window (90-360 min; red) (Mean and 90% credible interval)

| **Time window** | **Early (0-90 min)** | | **Late (90-360 min)** | |
| --- | --- | --- | --- | --- |
|  | **GLC [µM/min]**  Mean (90% CI) | **WP [µM/min]**  Mean (90% CI) | **GLC [µM/min]**  Mean (90% CI) | **WP [µM/min]**  Mean (90% CI) |
| ***v*_GLY_** | **21.29** (21.24-21.33) | **16.02** (15.93-16.11) | **0.175** (0.174-0.176) | **2.073** (2.054-2.092) |
| ***v*_LDHf_** | **53.72** (53.59-53.86) | **212.16** (210.62-213.70) | **3.335** (3.324-3.345) | **27.29** (27.03-27.55) |
| ***v*_LDHb_** | **42.38** (42.13-42.64) | **181.00** (179.39-182.60) | **1.701** (1.680-1.722) | **42.07** (41.38-42.76) |
| ***v*_LDH_net_** | **11.34** (11.12-11.56) | **31.17** (29.98-32.35) | **1.634** (1.613-1.655) | **-14.78** (-15.38-14.18) |
| ***v*_LDH_ex_** | **40.74** (40.53-40.95) | **166.95** (165.55-168.34) | **1.631** (1.612-1.649) | **21.25** (20.99-21.51) |
| ***v*_ALTf_** | **40.50** (40.26-40.74) | **175.42** (173.85-176.99) | **0.705** (0.693-0.717) | **6.275** (6.137-6.415) |
| ***v*_ALTb_** | **38.31** (38.05-38.56) | **192.04** (190.16-193.93) | **0.0150** (0.0146-0.0152) | **0.101** (0.098-0.103) |
| ***v*_ALT_net_** | **2.20** (2.04-2.36) | **-16.62** (-17.86-15.39) | **0.690** (0.679-0.702) | **6.174** (6.039-6.310) |
| ***v*_ALT_ex_** | **35.51** (35.28-35.74) | **155.95** (154.49-157.40) | **0.0149** (0.0146-0.0152) | **0.1007** (0.0983-0.1030) |
| ***v*_TCA_** | **1.65** (1.44-1.49) | **5.59** (5.51-5.67) | **0.0167** (0.0166-0.0168) | **0.0343** (0.0341-0.0346) |
| ***v*_pyr_** | **1.46** (1.44-1.49) | **3.06** (3.00-3.12) | **2.338** (2.320-2.357) | **22.62** (22.36-22.88) |
| ***v*_lac_** | **1.13** (1.12-1.15) | **2.45** (2.41-2.49) | **2.159** (2.140-2.178) | **46.42** (45.72-47.12) |
| ***v*_ala_** | **1.15** (1.13-1.17) | **3.08** (3.02-3.15) | **0.0038** (0.0037-0.0039) | **0.0264** (0.0255-0.0272) |
| ***v*_cit_** | **0.86** (0.85-0.87) | **2.97** (2.93-3.01) | **0.0066** (0.00659-0.00667) | **0.0414** (0.0411-0.0417) |

# References

[1] Hagström-Toft, E.; Enoksson, S.; Moberg, E.; Bolinder, J.; Arner, P. Absolute Concentrations of Glycerol and Lactate in Human Skeletal Muscle, Adipose Tissue, and Blood. *Am. J. Physiol.* **1997**, *273* (3 PART 1).

[2] FROSANDER, O. A.; RAEIHAE, N.; SALASPURO, M.; MAEENPAEAE, P. Influence of Ethanol on the Liver Metabolism of Fed and Starved Rats. *Biochem. J.* **1965**, *94* (February 1965), 259–265. https://doi.org/10.1042/bj0940259.

[3] Jansson, P. A.; Larsson, A.; Smith, U.; Lönnroth, P. Lactate Release from the Subcutaneous Tissue in Lean and Obese Men. *J. Clin. Invest.* **1994**, *93* (1), 240–246. https://doi.org/10.1172/JCI116951.

[4] Von Platen, A.; D’Souza, M. A.; Rooyackers, O.; Nowak, G. Evaluation of Intrahepatic Lactate/Pyruvate Ratio As a Marker for Ischemic Complications Early after Liver Transplantation-A Clinical Study. *Transplant. Direct* **2019**, *5* (12), 1–7. https://doi.org/10.1097/TXD.0000000000000952.

[5] Vesell, E. S.; Pool, P. E. Lactate and Pyruvate Concentrations in Exercised Ischemic Canine Muscle: Relationship of Tissue Substrate Level to Lactate Dehydrogenase Isozyme Pattern. *Proc. Natl. Acad. Sci. U. S. A.* **1966**, *55* (4), 756–762. https://doi.org/10.1073/pnas.55.4.756.

[6] Williamson, D. H.; Lopes-Vieira, O.; Walker, B. Concentrations of Free Glucogenic Amino Acids in Livers of Rats Subjected to Various Metabolic Stresses. *Biochem. J.* **1967**, *104* (2), 497–502. https://doi.org/10.1042/bj1040497.

[7] Maggs, D. G.; Jacob, R.; Rife, F.; Lange, R.; Leone, P.; During, M. J.; Tamborlane, W. V.; Sherwin, R. S. Interstitial Fluid Concentrations of Glycerol, Glucose, and Amino Acids in Human Quadricep Muscle and Adipose Tissue: Evidence for Significant Lipolysis in Skeletal Muscle. *J. Clin. Invest.* **1995**, *96* (1), 370–377. https://doi.org/10.1172/JCI118043.

[8] Røst, L. M.; Thorfinnsdottir, L. B.; Kumar, K.; Fuchino, K.; Langørgen, I. E.; Bartosova, Z.; Kristiansen, K. A.; Bruheim, P. Absolute Quantification of the Central Carbon Metabolome in Eight Commonly Applied Prokaryotic and Eukaryotic Model Systems. *Metabolites* **2020**, *10* (2). https://doi.org/10.3390/metabo10020074.

[9] Coggan, A. R.; Spina, R. J.; Kohrt, W. M.; Holloszy, J. O. Effect of Prolonged Exercise on Muscle Citrate Concentration before and after Endurance Training in Men. *Am. J. Physiol. - Endocrinol. Metab.* **1993**, *264* (2 27-2). https://doi.org/10.1152/ajpendo.1993.264.2.e215.

[10] Denton, R. M.; Halperin, M. L. The Control of Fatty Acid and Triglyceride Synthesis in Rat Epididymal Adipose Tissue. *Biochem. J.* **1968**.

[11] Gómez Perales, J. L. Blood Volume Analysis by Radioisotopic Dilution Techniques: State of the Art. *Appl. Radiat. Isot.* **2015**, *96*, 71–82. https://doi.org/10.1016/j.apradiso.2014.11.014.

[12] Östman, J. R.; Müllner, E.; Eriksson, J.; Kristinsson, H.; Gustafsson, J.; Witthöft, C.; Bergsten, P.; Moazzami, A. A. Glucose Appearance Rate Rather than the Blood Glucose Concentrations Explains Differences in Postprandial Insulin Responses between Wholemeal Rye and Refined Wheat Breads—Results from A Cross-Over Meal Study. *Mol. Nutr. Food Res.* **2019**, *63* (7), 1–9. https://doi.org/10.1002/mnfr.201800959.

[13] Boers, H. M.; Van Dijk, T. H.; Hiemstra, H.; Hoogenraad, A. R.; Mela, D. J.; Peters, H. P. F.; Vonk, R. J.; Priebe, M. G. Effect of Fibre Additions to Flatbread Flour Mixes on Glucose Kinetics: A Randomised Controlled. *Br. J. Nutr.* **2017**, *118* (10), 777–787. https://doi.org/10.1017/S0007114517002781

**
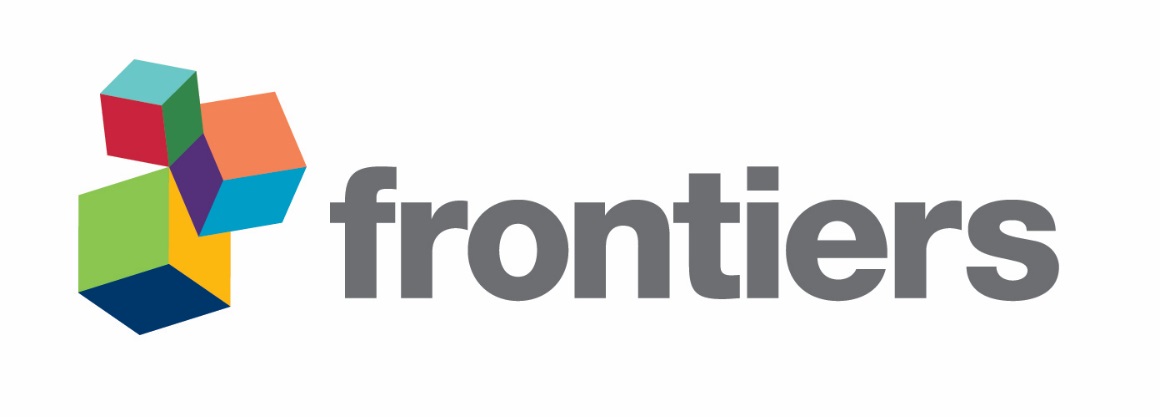
**
